# Selective Adsorption of Thiol-Containing Molecules on Copper Sulfide Surfaces via Molecule–Surface Disulfide Bridges

**DOI:** 10.1021/acs.jpcc.4c06463

**Published:** 2025-01-17

**Authors:** Connor R. Protter, Jennifer L. Bjorklund, Sara E. Mason, Robert J. Hamers

**Affiliations:** † Department of Chemistry, University of Wisconsin–Madison, 1101 University Avenue, Madison, Wisconsin 53706, United States; ‡ Center for Functional Nanomaterials, Brookhaven National Laboratory, Upton, New York 11973, United States

## Abstract

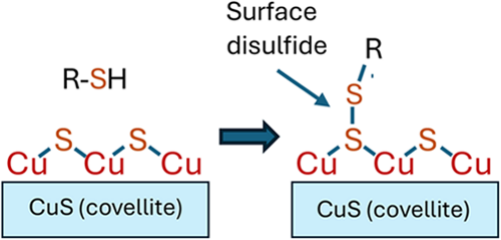

Recent results in the fields of nanoenhanced agriculture and expanding interest in prebiotic chemistry have placed increased emphasis on understanding the chemically selective interaction of small molecules with the surfaces of metal sulfides. We present an integrated experimental and computational study of the interaction of thiol-containing molecules with copper sulfide (covellite) surfaces in aqueous media. *In situ* Fourier-transform infrared (FTIR) measurements and *ex situ* X-ray photoelectron spectroscopy (XPS) measurements show that molecules bearing free thiol groups, including glutathione and cysteine, bind strongly to CuS (covellite) nanoparticles and to CuS (001) single crystals, while control studies show that similar molecules lacking the free thiol group exhibit much less binding. Additional experiments show that these thiol-containing molecules interact transiently with CuO nanoparticle surfaces but are readily removed by rinsing. The FTIR and XPS experiments demonstrate that adsorption of molecular thiols to CuS surfaces occurs in a chemically selective manner. Further experimental studies and density functional calculations show that the preferred mode of binding is through the surface S atoms, forming a Solid–S–S–Molecule disulfide linkage. While the role of disulfide linkages in controlling structure and function of proteins and other biomolecules is widely known, the formation of surface disulfide linkages as a motif for covalent molecular binding at surfaces has not been established previously.

## Introduction

Adsorption and chemical reactions at metal sulfide surfaces play an important role in the bioavailability of metals and the global geochemical cycling.^1−4^ The ability of iron sulfides and copper sulfides (including covellite, CuS) to catalyze the coupling of small-molecular precursors into nucleobases^5,6^ has stimulated great interest in understanding the possible role of metal sulfide surface interactions in prebiotic chemistry.^4,7^ Small CuS clusters have been found in the environment^8^ and are an integral part of some metalloenzymes such as cytochrome c oxidase.^9−11^ Here, the interaction of CuS clusters with adjacent S-containing residues to form disulfide bridges plays an essential role in enzyme function.^9−11^ These and other studies have motivated interest in understanding the surface chemistry of metal sulfides, and particularly the interaction of metal sulfides with thiol-containing molecules of relevance to biological chemistry.

Interest in copper-based nanoparticles has recently been further stimulated by studies showing that exposing plant leaves to CuO and Cu_3_(PO_4_)_2_ nanoparticles improves the plant’s innate defense mechanisms against root fungal diseases.^12−15^ The biological pathways stimulated by these nanoparticles are distinct from those produced by Cu^2+^ ions in solution (e.g., CuSO_4_ solutions),^13^ motivating a desire to understand the fundamental chemical properties that govern the interactions and transformations of Cu-containing nanoparticles.^15^ While the underlying physical and chemical pathways are not yet fully understood,^13^ prior studies have established that the behavior of nanoparticles in environmental and biological media is dictated by the adsorption of molecules from the surrounding matrix, forming a so-called “corona”.^16,17^ Yet, in most cases little is known about whether corona formation is controlled by chemically specific bonding interactions, or whether it is dominated by physical forces such as electrostatic interactions.^16−21^ These knowledge gaps arise because of the difficulty of probing adsorption and desorption processes on nanoparticle surfaces *in situ*.^16^

Here, we present results exploring the interactions of copper sulfide (covellite) surfaces with glutathione, cysteine, and related molecules as model systems for understanding the interaction of relevant biomolecules with copper-containing nanoparticles. Copper sulfide in the form of covellite is an important model system for study because Cu–S interactions are known to be important in Cu-containing biomolecules.^9−11^ Furthermore, covellite forms two-dimensional sheets much like CuO and Cu_3_(PO_4_)_2_, but the low solubility of CuS facilitates study of molecular adsorption processes in aqueous media with reduced complication from competing dissolution processes. Glutathione, a tripeptide, is widely distributed in animals, plants, and microorganisms^22−24^ and is one of the most widely prevalent thiols in biology, frequently found at concentrations of 0.1–10 mM^22−27^ In the present work we use *in situ* attenuated total reflectance-Fourier-transform infrared (ATR-FTIR) measurements to probe the vibrational features associated with adsorption and desorption from CuS nanoparticle surfaces in aqueous media and couple these measurements with *ex situ* X-ray photoelectron spectroscopy (XPS) studies to provide quantitative measures of molecular surface coverage. A comparison of glutathione with related molecules having specific changes in chemical structure reveals that the accessible thiol group leads to irreversible adsorption to CuS, while the amino, carboxylate, and disulfide linkages are less effective. In contrast to the irreversible adsorption on CuS, glutathione binds only transiently to CuO and is rapidly removed by rinsing. Detailed XPS studies on covellite single crystals under conditions maximizing surface sensitivity coupled with density functional computational studies and additional experimental studies indicate that this selective, irreversible adsorption of thiols to CuS occurs by forming disulfide bridges. While disulfide bridges play critical roles controlling the structure and function of biological systems, the possible formation of strong disulfide bridges in the form of a Solid–S–S−Molecule binding motif has been predicted computationally^28^ but not established experimentally. The coupling of *in situ* and ex *situ* experimental measurements with *ab initio* theoretical methods provides new insight into molecular factors controlling selectivity and irreversibility of molecule–surface adsorption. These insights contribute to a greater understanding of possible chemical approaches to control biological interactions of copper-containing nanoparticles.

## Materials and Methods

### Nanoparticle Synthesis

Ethanol (200 proof) was purchased from Decon Laboratories, Inc., and all other reagents were purchased from Sigma-Aldrich. We synthesized CuS nanoparticles following a modified procedure which has been reported on previously.^29^ Briefly, 0.7 mmol copper(II) acetate was mixed with 3 mmol sulfur in 100 mL ethanol and stirred for 10 min. The solution was then transferred to a Parr pressure vessel (Series 4760) and heated without stirring to 160 °C for 18 h before cooling overnight. This was centrifuged at 11,000*g* for 10 min to remove the supernatant, before being repeated a further three times with 18.2 MΩ·cm water. Particles were then dried overnight.

To compare how anion identity influences surface interactions of metal chalcogenides, we also conducted control experiments using CuO nanoparticles. CuO nanoparticle synthesis followed previously published procedures.^16^ Briefly, 1 mL of a 1 M CuCl_2_ solution was mixed with 2 mL of a 1 M LiOH solution, and diluted with 17 mL of deionized water. The mixture was stirred for 10 min and heated in a CEM Discover microwave synthesizer at 160 °C for 10 min. The particles were then cleaned via centrifugation as described above for CuS. We have previously reported detailed characterization of these CuO nanoparticles.^12,16^ Representative SEM images of the CuO nanoparticles used here (Figure S1) show that they consist of thin platelets identical in appearance to those we reported previously.

### Characterization of Nanoparticles

To prepare samples for scanning electron microscopy (SEM), dilute solutions of nanoparticles in isopropyl alcohol were sonicated and dropcast onto a conductive silicon wafer heated to 90 °C. Images were collected using a secondary electron detector on a Zeiss Supra55VP SEM. For powder X-ray diffraction (XRD), nanoparticles were packed in a zero-diffraction plate with a 0.2 mm well (MTI Corporation) and analyzed with a Bruker D8 Advance powder X-ray diffractometer from 20–80° with a step size of 0.1°.

X-ray photoelectron spectroscopy (XPS) data, unless otherwise specified, were collected on a Thermo K-α system using nanoparticles dropcast from an ethanol slurry onto boron-doped diamond substrates (Element 6) using a takeoff angle of 0° (i.e., collecting electrons emitted along the surface normal). Survey spectra were collected with a pass energy of 200 eV, 50 ms dwell time, and step size of 1 eV. High resolution spectra were collected with a 50 eV pass energy, 50 ms dwell time, and 0.2 eV step size. Detailed information on analysis using XPS can be found in the Supporting Information. Samples for ζ-potential analysis were prepared by sonicating a 50 mg/L solution of nanoparticles in deionized water for 10 min. ζ-potential was measured with a Malvern Zetasizer Nano ZS, and Brunauer–Emmett–Teller (BET) surface area was measured using a Micrometrics Gemini VII surface area analyzer.

Samples for Raman spectroscopy were prepared via dropcasting CuS nanoparticles from isopropyl alcohol onto a conductive silicon wafer heated to 90 °C. Spectra were collected from 200 to 3200 cm^–1^ on a Horiba LabRAM HR Evolution Raman Spectrometer with a 532 nm excitation source and Synapse BIDD CCD detector. Spectra reported here used a 10 s acquisition time and 10 accumulations.

### Molecules Selected for Analysis

**Scheme 1:**
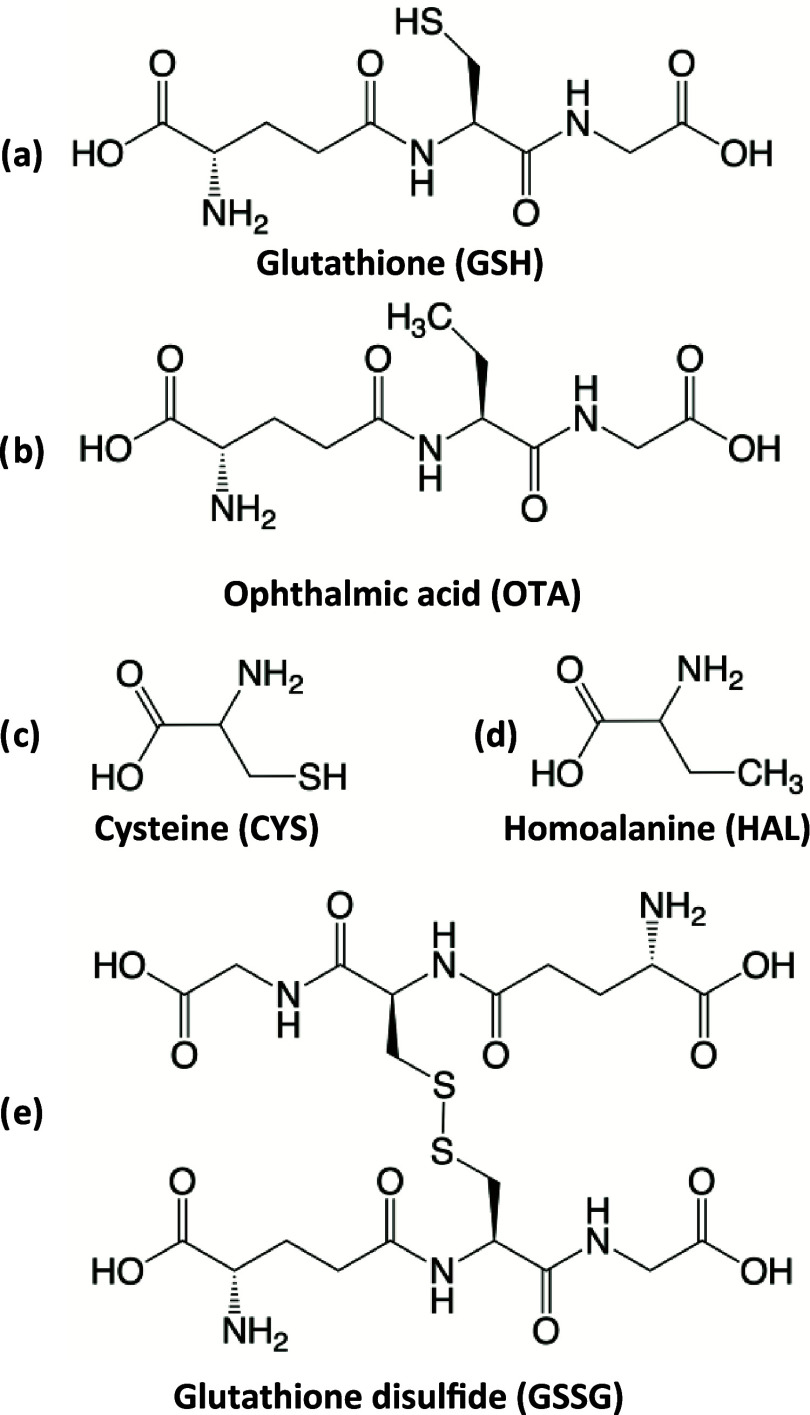
Model Molecule Names, Structures, and Abbreviations Used in This Study

Scheme 1 shows the selection of molecules used for this study alongside their structure and abbreviations. We chose glutathione as a model system because of its widespread presence in biological tissues.^30^ Other molecules were selected to establish the role of specific molecular functional groups on the interaction with CuS.

### *In Situ* Attenuated Total Internal Reflection-Fourier Transform Infrared (ATR-FTIR) Measurements

We first prepared 4 mL of a 1 mg/mL solution of nanoparticles in ethanol, dispersed with sonication. This was dropcast onto a germanium 10-bounce attenuated total internal reflection element (Specac, 75 mm long by 3 mm thick,) before drying at 400 °C for 4 min.

Spectra were collected on a Bruker Vertex 70 Fourier-transform Infrared (FTIR) spectrometer using a Specac Gateway ATR accessory with a 550 μL flow through top plate. In these experiments, we acquired FTIR spectra continuously (acquisition time of approximately 4 min per spectrum at 4 cm^–1^ resolution) throughout 3 steps: initial equilibration with pure water (30 min), exposure to molecule of interest (90 min), and pure water rinse (90 min). A constant 1 mL/min flow water was maintained throughout. Absorbances at various time points were calculated as  using the last spectrum of the initial rinse step as the reference spectrum *I*_ref_(υ̃). Control experiments using 2-mercaptoethanol were performed in a similar manner except that before rinsing, nanoparticles were exposed to a 5% w/w solution of 2-mercaptoethanol for 30 min. Unless otherwise specified, all solutions used in ATR-FTIR measurements were 65 μM concentration.

### X-ray Photoelectron Spectroscopy

Samples were prepared by dropcasting a nanoparticle slurry onto boron-doped diamond, and sealing this substrate into a flow cell to mimic the conditions used in the ATR-FTIR experiments. Using an NE-1000 syringe pump (New Era Pump Systems, Inc.) dropcast nanoparticle films were equilibrated with water, exposed to model molecules, and rinsed following the same procedures as above for ATR-FTIR studies. XPS exposure experiments used a flow rate of 0.2 mL/min, yielding a linear flow velocity similar to that of the ATR-FTIR studies. For the 2-mercaptoethanol exposure, the process was the same as above, except that the sample was additionally exposed to a 5% w/w aqueous solution of 2-mercaptoethanol prior to rinsing. These samples were then dried for analysis. Instrumental parameters for these experiments are the same as described above. Statistical analysis was conducted using JMP, Version 17.2.0 (SAS Institute Inc., Cary, NC).

The sampling depth of XPS is controlled by the inelastic mean free path (typically ∼2 nm) of the electrons that are emitted and the angle at which electrons are collected. Collecting electrons emitted at very shallow angles with respect to the surface plane yields improved sensitivity to the very outermost region of the sample, improving the ability to probe the surface chemistry of importance to our studies. This approach requires samples with exposed faces that are flat and several mm in size so that the electron emission angles are well-defined with respect to the surface plane. Since covellite samples meeting these requirements are not readily synthesized in the laboratory, we used natural single-crystal covellite samples (Excalibur Mineral Corporation). These were cleaned with 12 M HCl prior to use. Covellite was exposed to 65 μM glutathione on an orbital shaker for 1 h before rinsing. XPS measurements using takeoff angles of 80° from the surface normal were conducted using a Phi VersaProbe III system. To achieve a well-defined takeoff angle, we rotated the sample appropriately and introduced an aperture between the sample and the analyzer. High-resolution spectra were collected with a 55 eV pass energy, 50 ms dwell time, and 0.1 eV step size. Details of energy calibration and peak-fitting are presented in the Supporting Information.

### Covellite Dissolution Studies

To determine the impact of molecular adsorption on the release of copper from CuS, we carried out dissolution studies in triplicate using two solution sets: a “plant-like” medium containing 890 μM malic acid and 1.71 mM citric acid, and a second medium containing those with the addition of 1 mM glutathione. Solutions were removed after 2, 8, 24 h, and 4 days, before being centrifuged at 13,100*g* (Eppendorf MiniSpin Plus) for 10 min, and filtered through 0.1 μm syringe filters. Solutions were then acidified with 2.5% HNO_3_ and analyzed by ICP-MS (8900 Triple Quadrupole, Agilent) with yttrium as an internal standard.

### Computational Methods

To understand the structure of CuS surfaces and the thermodynamics associated with adsorption of cysteine as a model thiol, we used *ab initio* atomistic thermodynamics calculations. Cysteine was chosen over glutathione to reduce the number of possible molecular conformations. A detailed explanation of the underlying methodology is contained in the Supporting Information. Briefly, periodic density-functional theory (DFT) calculations were performed on model CuS structures using the generalized gradient approximation of Perdue, Burke, and Ernzerhof (PBE) as implemented within the DMol^3^ code.^31,32^ All-electron calculations were done using a double numeric basis set with polarization (DNP).^33^ All structures were subject to full geometry optimizations, where self-consistent field energies were converged to at least 3 × 10^–6^ eV, and the optimization reached a threshold of at least 3 × 10^–5^ eV or until the force on each atom converged to <1 meV/Å. Molecular calculations were also carried out with the use of periodic boundary conditions, with O_2_ and H_2_ molecules described in the gas phase. H_2_O and cysteine molecules were modeled with the use of the implicit solvation model COSMO, wherein the calculated gradients include forces between the solute and screening charges to simulate the electrostatic effects an aqueous environment with a dielectric constant (ε) of 78.54.^34,35^

The bulk covellite CuS structure, space group *P*6_3_/*mmc* (194) was modeled in a hexagonal unit cell using a Monkhorst–Pack^36^ 8 × 8 × 4 *k*-grid, yielding optimized lattice constants of *a* = *b* = 3.838 Å and *c* = 16.583 Å, in reasonable agreement with experimental values (*a* = *b* = 3.794 Å and *c* = 16.341 Å) and previously reported theoretical values.^37−40^ The *k*-grid is folded down to 6 × 6 × 1 for the optimization of bare and hydrated surface slabs.

**Figure 1:**
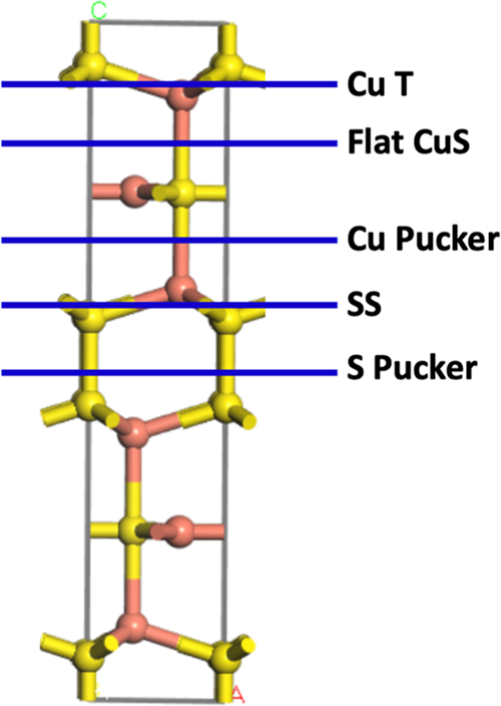
Side view of the CuS unit cell, where the Cu and S atoms are represented in ball-and-stick form as orange and yellow balls, respectively. The five possible bond cleavage planes are identified and labeled, where atoms above the blue line are removed to create the surface slab and the name refers to the terminating atom.

As shown in Figure 1, sulfur has two chemically distinct sites, comprised of Cu–S–Cu (“sulfide”) and Cu–S–S–Cu layers (“disulfide”) layers. Prior studies of single-crystal covellite,^41^ nanocrystalline covellite prepared through different synthesis routes,^42−44^ and computational studies of covellite thermodynamics^37,40^ have uniformly concluded that the (001) plane has the lowest surface energy and is the predominant face exposed. SEM images of our nanoparticles (*vide infra*) show thin platelets having almost perfect hexagonal symmetry. We therefore focused our studies on the (001) crystal face and the different possible surface terminations. The optimized bulk CuS was cleaved along the (001) direction to generate the 1 × 1 surface models, where the different bond cleavage planes are identified in Figure 1. There are five possible surfaces that can be formed that exhibit different terminating atom(s): two S-terminated (SS and S Pucker), two Cu-terminated (Cu T and Cu Pucker), and one CuS-terminated (Flat CuS). All surface slab models were set up with inversion symmetry; in doing so, any polarization effects caused by the cleaving process are negated. Slab thicknesses ranged between 13–20 Å, with at least 15 Å of vacuum between periodic slabs to avoid through-space interaction of periodic repeats.

## Results and Discussion

### Nanoparticle Morphology, Crystallinity, and Surface Properties

**Figure 2:**
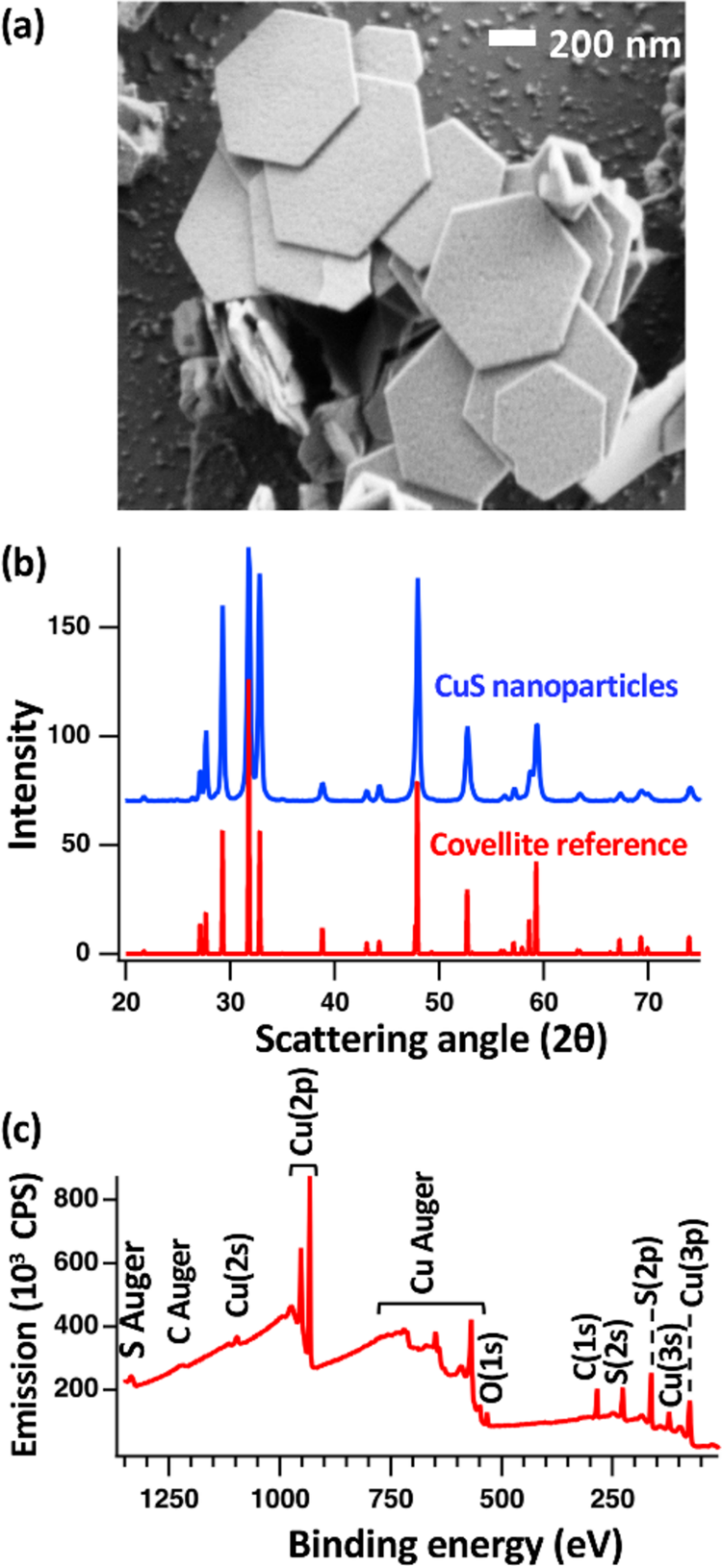
Characterization of bare CuS nanoparticles, including (a) representative electron micrograph, (b) X-ray diffraction pattern, and (c) XPS survey data.

Figure 2 shows characterization of the CuS nanoparticles using SEM (Figure 2a), XRD (2b), and XPS (2c). The SEM image in Figure 2a image shows that the nanoparticles consist of thin platelets with hexagonal habit, with outgrowths distributed across some particles. The anisotropic habit and hexagonal shape indicate that the broad faces expose the (001) plane, in agreement with previous experimental and computational studies.^41−44^ Detailed analysis of SEM images using ImageJ software show that the average nanoparticle size, measured by the distance between opposite edges of the hexagons, is 500 ± 130 nm (*N* = 258) with a thickness of 70 ± 20 nm (*N* = 167). Figure 2b shows powder X-ray diffraction data from our CuS nanoparticles, along with a reference spectrum for the CuS covellite structure.^45^ Our experimental data are in excellent agreement with the reference spectrum with no traces of other structures, thereby indicating a high degree of structural purity. Figure S2 includes a comparison showing that the XRD features match that of a reference covellite spectrum and are distinct from other copper sulfide phases. Figure 2c shows XPS survey spectra of the nanoparticle surfaces.

Analysis of fractional surface composition (details in Supporting Information (SI)) yields a Cu:S ratio of 1.08:1 and is consistent with that of the 1:1 stoichiometric ratio for bulk covellite. We additionally performed more detailed analysis of the nanomaterial surfaces using high-resolution XPS and by Auger parameter analysis. Detailed analysis of the S(2p) region by XPS shown in Figure S3 reveals three set of peaks that are attributed to S atoms in the Cu–S–Cu layers (161.2 eV) and the Cu–S–S–Cu layers (162.0 eV), along with a third feature at higher binding energy (163.0 eV). This feature can likely be attributed to a combination of shakeup features and chemically distinct surface species.^46−50^ Previous studies have shown that the modified Auger parameter, the difference in energy between Auger and photoelectron peaks for specific elements, is distinct for various copper compounds.^51^ We performed a modified Auger parameter analysis by summing the binding energy of the Cu(2p_3/2_) photoelectron and kinetic energy of the L_3_M_45_M_45_ peak from the XPS survey in Figure 2c, and found a value of 1850.5 eV. This value is within the accepted range of modified Auger parameter values for CuS of 1850.3 ± 0.2 eV for covellite and is significantly greater than the value anticipated for other stoichiometric and nonstoichiometric copper sulfides.^51−53^

We further characterized our CuS nanoparticles using Raman spectroscopy. Figure S4 shows that the Raman spectrum is in close agreement with that expected for covellite and is distinct from other copper sulfides. ζ-potential measurements show a ζ-potential of −27 ± 1 mV, and the BET surface area of these nanoparticles is 11.0 m^2^/g.

### Adsorption of Glutathione Is Surface-Selective between CuS and CuO

**Figure 3:**
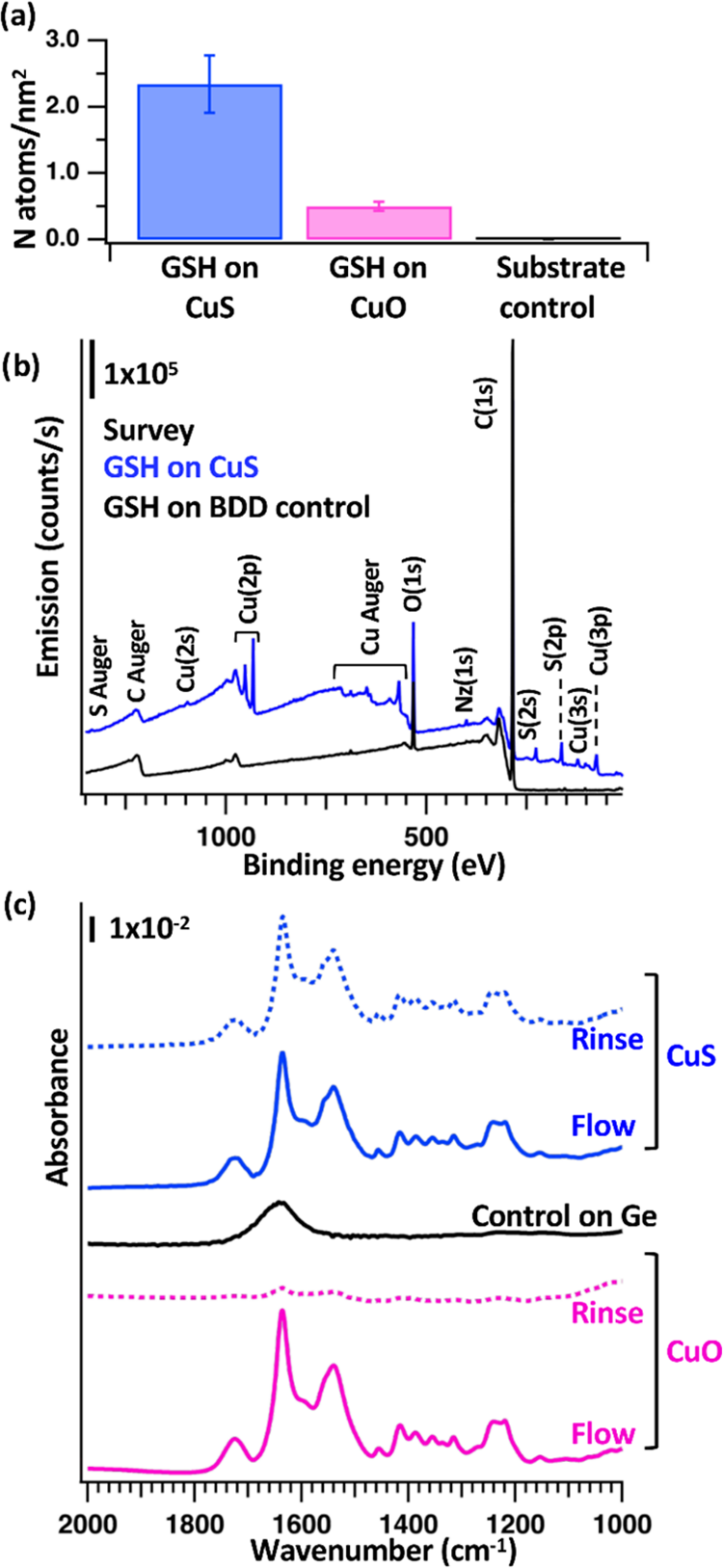
Characterization of glutathione adsorption (a) Nitrogen surface coverage after CuS nanoparticles, CuO nanoparticles, and boron-doped diamond substrate (as a control) were exposed to GSH. (b) Representative XPS spectra from GSH on CuS nanoparticles and BDD substrate control. (c) *In situ* FTIR measurement of GSH interaction with CuS and CuO nanoparticles. The black trace represents glutathione flowed over the bare Ge internal reflection element.

Figure 3 shows data probing the interaction of glutathione with CuS and CuO surfaces using XPS and FTIR. We used N(1s) emission intensity to quantify the molecular coverage of glutathione on these surfaces, using the Cu(2p) emission as an internal standard. From the areas of the N(1s) (Figure S5) and Cu(2p) regions, in conjunction with the inelastic mean free paths and the atomic sensitivity factors, we estimated the absolute surface coverage as shown in Figure 3a. Details of this analysis are described in the Supporting Information. Figure 3b shows XPS survey spectra of a CuS sample that was equilibrated with pure water, exposed to glutathione, and then rinsed for 90 min as described above. In this experiment, the CuS nanoparticles were dropcast onto a conductive boron-doped diamond substrate. The XPS data show that glutathione adsorbs strongly to CuS surfaces, yielding molecular coverages of ∼2 molecules/nm^2^. In contrast, interactions with CuO shows much lower coverage, only ∼0.5 molecules/nm^2^. Statistical analysis shows that the difference in adsorption on CuS and CuO surfaces is significant at the 95% confidence interval. While the extent of adsorption can be impacted by many factors, ζ-potentials of CuS (−27 ± 1 mV) and CuO (−19.7 ± 0.3 mV) are similar. Thus, we conclude that the difference in binding affinity of glutathione on CuS vs CuO is likely not a result of electrostatic interactions but is more likely due to a chemically specific interaction.

While XPS provides information about the total extent of adsorption, we used *in situ* FTIR spectroscopy to probe the changes in vibrational spectra during exposure to glutathione and during the subsequent rinse. As described above, each *in situ* experiment generates spectra at multiple time points, yielding both spectroscopic and kinetic information. In the Supporting Information Figure S6, we show a complete set through a typical 90 min exposure and 90 min rinse. The *in situ* spectra show vibrational features due to interaction of glutathione with the surfaces, plus additional features arising from atmospheric water vapor, atmospheric CO_2_, and imperfectly compensated liquid water. Examination of the compete set shows that changes in the vibrational spectra during the exposure step and during the rinse step are each complete within tens of minutes. We therefore focus attention on the last spectrum obtained while flowing glutathione (“flow”) and the last spectrum of the subsequent 90 min rinse (“rinse”) for more detailed analysis, shown in Figure 3c. These spectra are limited to the amide vibrational region and have been background-subtracted by removing broadly sloping backgrounds. Figure 3c also shows an infrared spectrum of glutathione measured using the same ATR crystal, but without the CuS nanoparticles present. We note that to obtain a spectrum with comparable absorbance, it was necessary to go to concentrations approximately 1000-fold higher (100 mM) compared to the spectra obtained with the CuS nanoparticles (65 μM). We therefore conclude that the vibrational features observed in the *in situ* spectrum at 65 mM concentration arise almost exclusively from glutathione adsorbed to the CuS nanoparticles surfaces, with only negligible contribution from glutathione in the aqueous phase.

To glean more information the glutathione-CuS interaction, we examined the relative intensity of the peaks associated with glutathione in solution and bound to the surface. The absorbances shown in these spectra match well with those for glutathione, including a carboxylic acid C=O stretch at 1725 cm^–1^, peaks associated with amides at 1636 and 1540 cm^–1^, and further complex amide bands between 1500–1100 cm^–1^.^54,55^ We were not able to observe the S–H vibrational modes of glutathione either in the presence of or absence of CuS nanoparticles; this result is consistent with prior reports that thiol group vibrations are frequently very weak and broad due to hydrogen bonding.^56^ Our FTIR spectra are consistent with the XPS data, showing that adsorption of glutathione occurs quick (within tens of minutes) and irreversibly, as there is no significant decrease in amide band intensity when the 65 mM glutathione is replaced with deionized water for 90 min.

As a comparison, we also performed similar ATR-FTIR samples using CuO nanoparticles instead of CuS nanoparticles. Figure 3 shows that with CuO nanoparticles, the FTIR spectra obtained while flowing GSH show significant FTIR intensity, but the features disappear after the GSH is replaced with deionized water. During flow, the amide band intensity is again much larger than what would be expected from the solution-phase GSH alone. Therefore, we conclude that with CuO surfaces, glutathione binds weakly to the surface and is concentrated on the surface while GSH is flowing, but the surface-bound molecules are easily removed when the GSH-containing solution is replaced with pure water.

From the above experiments, we conclude that glutathione binds to CuS surfaces within several minutes of exposure and that this binding is irreversible on the ∼90 min time scale investigated here. Glutathione also interacts with CuO when excess GSH is present in the aqueous phase but desorbs rapidly during the water rinse. These observations are consistent with the XPS results but provide additional insight into the adsorption and desorption processes.

### Influence of Adsorbate Molecular Structure

The above results show that glutathione interacts strongly with CuS but not with CuO. To determine whether this interaction is specific to the presence of thiol groups or not, we performed similar experiments using a set of model molecules as depicted in Scheme 1. As a control for the adsorption of glutathione, we used ophthalmic acid (OTA), an analogous tripeptide in which the thiol group is replaced with a methyl group. We also performed experiments with the small amino acids cysteine (CYS) and homoalanine (HAL) to probe the influence of molecular size. Finally, we used glutathione disulfide (GSSG) to probe the importance of free thiol groups. We characterized the molecular interactions using XPS and FTIR in a manner similar to the experiments using glutathione.

**Figure 4:**
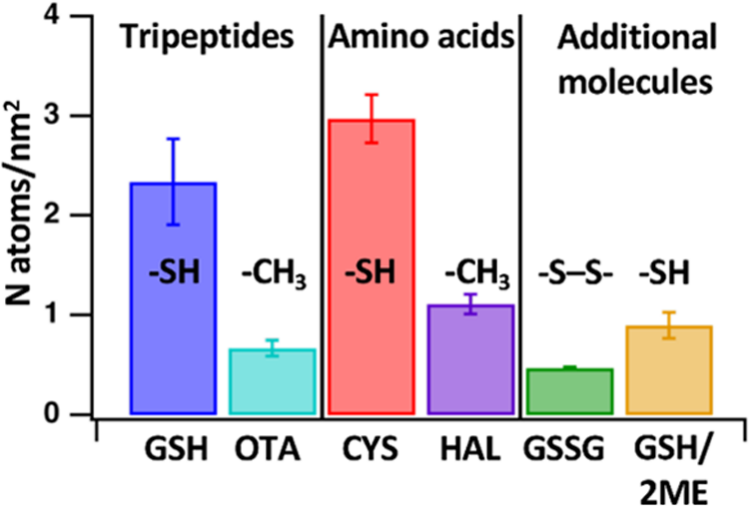
Comparison of surface coverage for additional molecules on the surface of CuS nanoparticles determined by XPS.

Figure 4 shows the XPS surface coverages of the molecules described above. We again use N(1s) emission to evaluate the surface concentration of adsorbed molecules. The surfaces exposed to glutathione and those exposed to cysteine show significant surface N concentrations. In contrast, surfaces exposed to homoalanine, glutathione disulfide, and ophthalmic acid exhibit N concentrations that are significantly lower from glutathione and cysteine concentrations at the 95% confidence interval. Quantifying the coverage using the Cu(2p) emission as an internal standard shows that molecules with accessible thiol groups (glutathione and cysteine) yield high surface coverages, while molecules with similar structures but lacking the thiol group (i.e, homoalanine, glutathione disulfide, and ophthalmic acid) yield much lower surface coverages.

Our data suggests that surface binding of thiol-containing molecules occurs via formation of surface disulfide linkages. Disulfide bridges are ubiquitous in biology and are present in one-third of eukaryotic proteins.^57^ In biological chemistry, exposure to 2-mercaptoethanol, which cleaves disulfide linkages, is widely used as a test for the presence of disulfide linkages. To probe this, we performed an experiment in which a CuS nanoparticle film was exposed to glutathione as in other experiments, and then the sample was rinsed with a solution containing 2-mercaptoethanol (2ME). The XPS data show that exposure of the GSH-exposed surface to 2-mercaptoethanol significantly decreases the surface N coverage, implying that 2ME cleaves most (but not all) of the surface-molecule bonds.

**Figure 5:**
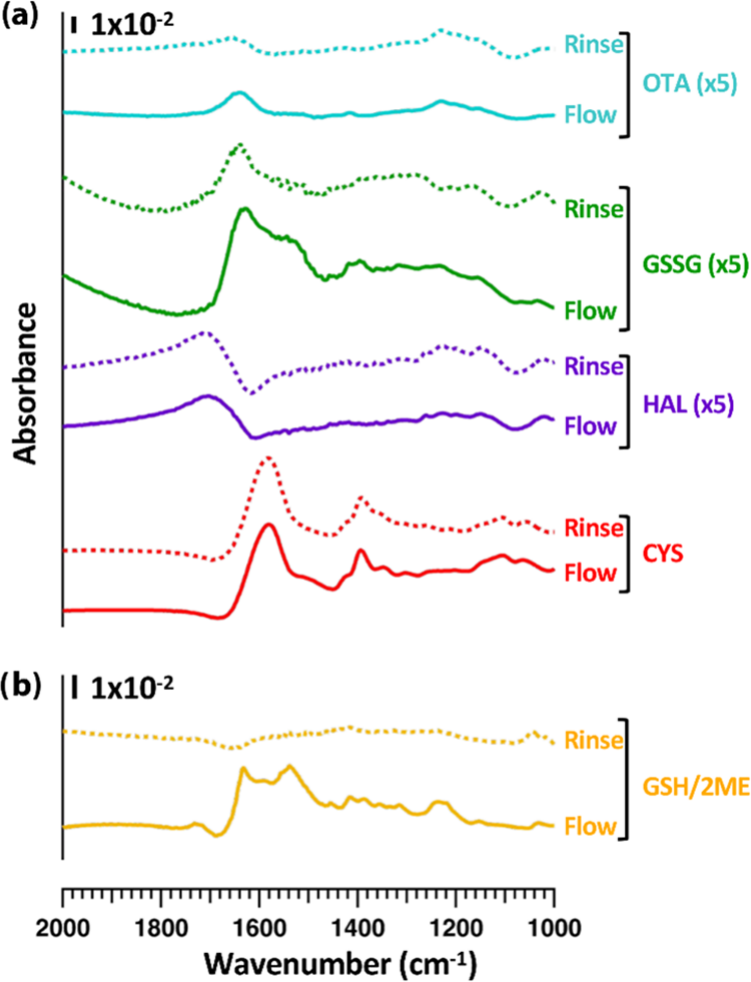
Infrared spectra. (a) Absorbance spectra of CuS surfaces while flowing solutions of the indicated molecules (“Flow”) and while rinsing with deionized water (“Rinse”). In each case the spectra shown correspond to the last spectrum obtained during a 90 min exposure to the indicated molecule and the last spectrum obtained during a subsequent 90 min rinse with deionized water. (b) Spectrum of GSH-exposed surface and same sample after 30 min rinse with solution containing 2-mercaptoethanol (2ME).

Figure 5a shows FTIR spectrum for CuS nanoparticle films exposed to each of these molecules. In each case, the *in situ* experiments show rapid (tens of minutes) adsorption, with little or no decrease in absorbance when the flowing solutions are replaced with pure water rise. The spectra for ophthalmic acid, glutathione disulfide, and homoalanine have been magnified 5-fold due to the low intensity of peaks associated with adsorption for each of these molecules. The spectra in Figure 5a fully corroborate the XPS data for each of these molecules, showing that cysteine and glutathione, both of which have accessible thiol groups, adsorb strongly, while ophthalmic acid, glutathione disulfide, and homoalanine do not. Figure 5b shows *in situ* FTIR data for of a sample that was exposed to GSH, and then the same sample after flowing 2-mercaptoethanol (2ME) over it. In agreement with the XPS data above, the FTIR data show that exposing the surface-adsorbed GSH molecules to 2ME cleaves the GSH molecules from the surface.

To confirm that the chemical selectivity inferred from the above data, we performed two additional sets of control experiments. Measurements of the IR spectra of the different molecules using a single-bounce Ge ATR element in the absence of any nanoparticles (Figure S7, using 100 mM concentration) show that the molecules in solution all have IR absorption features with similar intensities. This indicates that the molar absorptivities, and therefore the analytical sensitivity to each molecule in solution, are comparable to one another. The data in Figure S7 were obtained at much higher concentration (100 mM) compared to those used elsewhere in this work (65 μM). Figure S8 shows IR spectra obtained on the multiple-bounce Ge ATR element, again in the absence of nanoparticles, at the 65 μM concentration used in other experiments reported here. These spectra show much lower absorbances compared to those in Figure 5; this large difference indicates the contributions to the spectra in Figures 3 and 5 from free molecules in solution are negligible relative to those adsorbed on the surface.

### XPS with Maximum Surface Sensitivity

The presence of internal disulfides as an integral part of the covellite crystal lattice complicates detection of possible disulfides forming at the CuS–molecule interface. In an effort to isolate the spectroscopic features of the glutathione–CuS interfacial bonds, we used high-angle XPS to maximize the surface sensitivity and therefore specificity of our measurements. If a planar sample is tilted such that only electrons emitted close to the surface plane can reach the analyzer, electrons arising from the bulk are preferentially scattered and reduced in intensity compared to those from the outmost surface. Collecting electrons emitted at 80° from the surface normal (compared to 0° used elsewhere in this work) yields an improvement of ∼6× in sensitivity to the surface compared to the near-surface bulk region.

**Figure 6:**
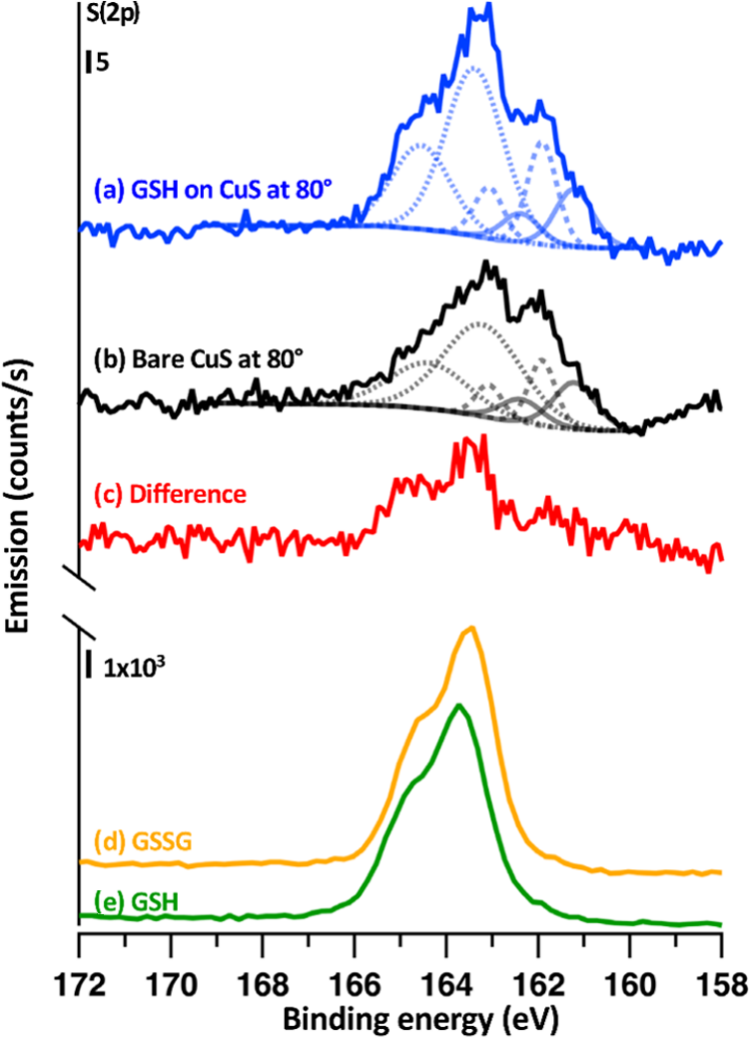
XPS spectra using 80° takeoff angle of (a) CuS exposed to glutathione, (b) bare CuS, ((c) b subtracted from a). Also shown are XPS spectra of thin films of (d) glutathione disulfide and (e) glutathione. Angles indicated are relative to the surface normal.

Figure 6 shows S(2p) XPS spectra acquired using electron escape angle of 80° from surface normal for the same covellite single-crystal sample, both before and after exposure to glutathione. Peak-fitting using procedures identical to those used in Figure S3 yielded 2p_3/2_ peaks at 161.2, 161.9, and 163.2 eV, along with their respective 2p_1/2_ components shifted by 1.18 eV. These energies are nearly identical to those in Figure S3. Peak-fitting for these components resulted in a reduced χ^2^ = 1.12, indicating that the residual error between data and fit is only slightly greater than that attributable to the noise.

The glutathione-exposed sample spectrum shows an increase in the 2p_3/2_ feature at higher binding energy shifting slightly to 163.4 eV. As noted earlier, broad emission in this range on the starting surface can arise from shakeup features and from surface species. In order to isolate the glutathione-induced changes without additional peak fitting, we determined the S(2p) changes upon molecular adsorption by direct subtraction of the starting surface from the glutathione-exposed sample. This difference spectrum shows two distinct peaks, at 163.4 and 164.6 eV, with a 2:1 intensity ratio. The 1.2 eV splitting and 2:1 intensity ratio are both consistent with these peaks arising from the 2p_3/2_ and 2p_1/2_ spin–orbit of a single chemical form of sulfur. While the precise structure of the exposed CuS surface remains unknown, a qualitative analysis of the S(2p) region provides some insight. Compared with neutral S atoms, S atoms in bulk CuS are in a more ionic environment; the electronegative S atoms withdraw electron density from Cu, leading to partial negative charges on the S atoms and core electrons that are more weakly bound. Thus, the 2p_3/2_ binding energy of S atoms bound to nearby Cu is 161.2 eV, compared with approximately 163 eV for polysulfides with S atoms in a nearly neutral environment.^58−60^ The new feature we observe after glutathione exposure has a binding energy close to that of the neutral polysulfides, suggesting that that S atom of glutathione is binding to another S atom of the surface rather than an exposed Cu^2+^ ion.

Our XPS and FTIR data suggest that glutathione, cysteine, and presumably other thiols also bind to CuS surface via formation of surface disulfide linkages. We performed density functional theory calculations to assess whether this type of bonding motif is feasible thermodynamically.

### DFT + Thermodynamics: Surface Functional Groups

**Figure 7:**
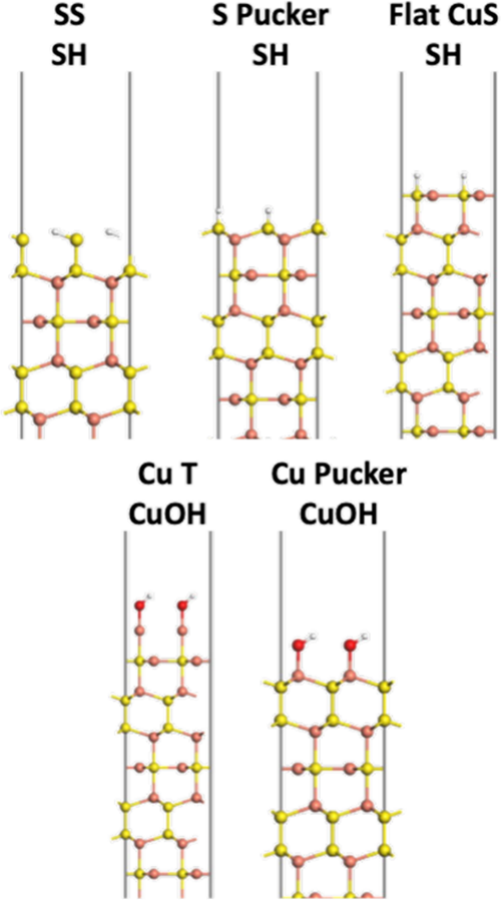
Side views of five possible surface terminations on the CuS surface under hydrating conditions. Atoms are represented using ball-and-stick models, where Cu, S, O, and H atoms are colored orange, yellow, red, and white, respectively.

To explore the overall thermodynamic stability of binding via surface dithiol linkages vs other possible bonding configurations, we used DFT calculations of CuS surfaces both in the presence and absence of cysteine as a model adsorbate. These calculations included the influence of water on the surface termination and the dielectric constant of the medium. Figure 7 compares the different functional groups that can form on the CuS covellite (001) surface when hydrated. We expect that surface sites terminated with undercoordinated S atoms will protonate to form thiol (SH) functional groups and that surface sites exposing under-coordinated Cu atoms will hydroxylate to form copper hydroxyl (CuOH) functional groups. Cleavage along a (001) plane can produce surfaces with several different terminations depending on the precise location of the exposed atomic layer. Detailed explanation of the DFT + Thermodynamics methodology is contained in the Supporting Information, but the overall reactions used in the formation of SH and CuOH functional groups are shown in eqs 1 and 2, respectively.
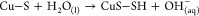
1
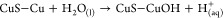
2

Previous work characterizing aqueous metal oxide minerals has demonstrated the tendency for surface functionalization to influence the thermodynamic stability of different facets and termination schemes.^61−64^ The values of Δ*G*_total_ for the formation of SH and CuOH functional groups are presented below in Table 1. A positive Δ*G*_total_ value indicates that the formation of surface functional groups destabilizes the surface, while a negative value indicates increased stabilization. All Δ*G*_total_ values range ±1 eV, with the largest value being +0.89 eV for the formation of SH groups on Flat CuS and the smallest value being −0.91 for the formation of CuOH groups on the Cu T surface. There are three surfaces that exhibit favorable Δ*G* values: Cu T (CuOH), SS (SH), and S Pucker (SH). As such, only these three surfaces are considered as models for biomolecule adsorption studies.

**Table 1:** Calculated Values of Δ*G* Using the DFT + Thermodynamics Approach for the Formation of Five Possible Surface Terminations

termination	Cu T	Cu Pucker	flat CuS	SS	S Pucker
functional group	CuOH	CuOH	SH	SH	SH
Δ*G*_1_ (eV)	–2.96	–1.58	+0.48	–0.51	–0.71
Δ*G*_2_ (eV)	+2.05	+2.05	+0.41	+0.41	+0.41
Δ*G*_total_ (eV)	–0.91	+0.47	+0.89	–0.09	–0.30

### Computational Modeling of Cysteine Adsorption

Using the previously determined stable SH terminated structures (SS and S Pucker), 2 × 2 supercells were generated to model adsorption at reasonable coverage. Cysteine adsorption was modeled as an analog to glutathione to reduce the computational cost and limit the number and types of surface functional groups that could possibly engage the CuS surface, and to test the thermodynamic likelihood of S–S bond formation at the CuS surface. Inner-sphere adsorption on the OH-terminated Cu T surface was deemed unfavorable as no stable adsorption geometry could be identified without the surface and/or adsorbate undergoing significant structural changes that cannot be directly compared to the SH-terminated surfaces. Details of the mechanisms modeled here can be found in the Supporting Information.

**Figure 8:**
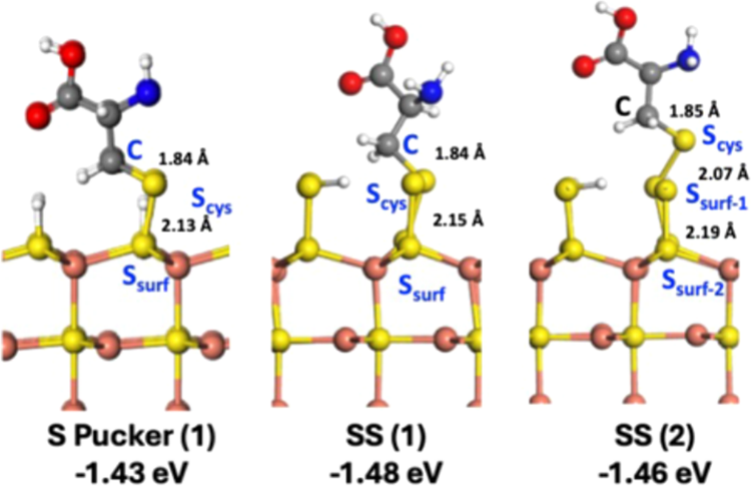
Optimized geometries of cysteine-CuS adsorption complexes and their respective *E*_ads_ values. The number in parentheses indicates which reaction scheme was used to compute *E*_ads_. Atoms are represented using ball-and-stick models, where the Cu, S, O, H, C, and N atoms are colored orange, yellow, red, white, gray, and blue, respectively.

The optimized adsorption geometries are shown in Figure 8 for the three possible structures; the *E*_ads_ values for the formation of each complex is indicated below. We note that the *E*_ads_ values for the formation of all three complexes are all favorable and very similar, ranging only by 0.05 eV regardless of if the surface terminates with SS or S Pucker. Comparing the two structures that bind cysteine to the surface through S–S bonds (labeled with (1)), both adsorption complexes have similar C–S_cys_ bond lengths to the optimized isolated cysteine molecule, but the S Pucker (1) structure has slightly smaller S_cys_–S_surf_ bonds compared to the SS (1) complex. This slight difference in the one bond length results in a smaller S_surf_–S_cys_–C bond angle of 104.4° for the S Pucker (1) adsorption geometry compared to the SS (1) angle of 110.6°.

Considering the SS (2) structure, the S_surf-2_–S_surf-1_ bond of SS (2) is more like the S_surf_–S_cys_ bonds of SS (1) and Pucker (1) than the S_surf-1_–S_cys_ bond. The formation of S–S–S bonds causes lengthening of the S_cys_-S_surf-1_ bond compared to the S_cys_–S_surf_ bonds compared to the (1) structures. The C–S_cys_ bond in SS (2) is also slightly longer but seems to be less sensitive to changes in local environment than the S_cys_–S_surf_ bonds. The S_surf-1_–S_cys_–C bond angle is 103.6°, which is smaller than the (1) analogs, due to the short S_surf-1_–S_cys_ bond length. It is also worth noting that the formation of a trisulfide bond, shown as SS (2), is just as favorable as formation of the disulfides in S Pucker (1) and SS (1). Given that sulfur is known to form polysulfides favorably,^65^ this is not an entirely unexpected result, although the similar *E*_ads_ values for each interaction in the CuS-cysteine system means that experimental verification is difficult.

DFT calculations were also performed to assess the vibrational frequencies of anticipated surface species. The results, shown in Supporting Information, indicate that the vibrational modes of the S–S surface species are predicted to be very close to those of the bulk sulfur-related Cu–S–S–Cu modes. This is consistent with experimental efforts that we made to detect surface species by Raman spectroscopy of the GSH-exposed surface, which were unsuccessful due to the large background from the bulk S–S modes.

### Influence of Glutathione Adsorption on Cu Release in Aqueous Media

Previous studies of nanoparticles in aqueous media and biological systems have shown that the composition of the aqueous matrix can significantly impact the rates of dissolution and other transformations. Small organic acids, especially citric acid, have been shown to play an important role^66−68^ and are naturally present in the at millimolar concentrations in many plant species^69−71^ and have been used in simulated xylem media for studies on agriculturally relevant nanomaterials.^12,72^ In order to identify how copper release is affected by the adsorption of glutathione, we investigated dissolution of Cu into two different “plant-like” aqueous media: one containing 890 μM malic acid and 1.71 mM citric acid at pH 6, and one containing both of those alongside 1 mM glutathione.

**Figure 9:**
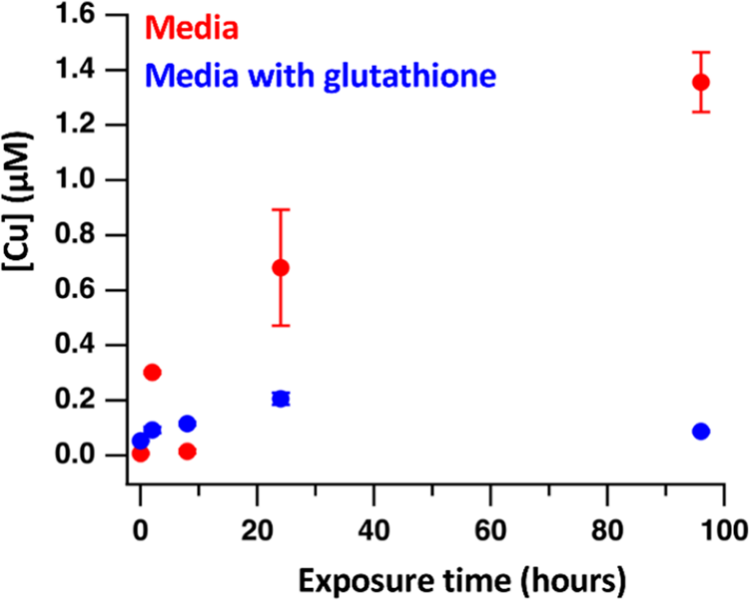
Comparison of Cu release from CuS nanoparticles at multiple time points in “plant-like” media, compared to the same media with added glutathione, measured by ICP-MS.

Figure 9 shows ICP-MS Cu data for nanoparticles exposed to the aforementioned sample media for durations of 2, 8, 24, and 96 h, alongside a media blank containing no nanoparticles (shown at time point 0). The particles in typical “plant-like” media release a small concentration of Cu^2+^, yielding concentrations of approximately 1.3 μM Cu^2+^ after 96 h. Interestingly, however, nanoparticles exposed to the media containing glutathione results in very little Cu release (0.1 μM). Thus, we conclude that the high coverage of glutathione effectively thwarts dissolution and release of Cu^2+^, even in the presence of chelating weak acids.

## Conclusions

The work reported here shows that thiol groups of small molecules of biological significance bind irreversibly to covellite surfaces, while other common functional groups (e.g., carboxylate groups and amino groups) induce much weaker interactions. This conclusion is consistent with previous reports that that cysteine-containing peptides have particularly strong affinities for binding to ZnS.^73^ Our experimental and computational results both point to the formation of molecule–surface disulfide linkages in which the free thiol group of the molecule binds to a sulfur atom of the CuS lattice as depicted in Figure 8 as the surface binding motif. This conclusion is consistent with a prior molecular dynamics study of amino acid interactions with ZnS.^28^ That study^28^ calculated that cysteine formed a covalent bond to ZnS via a disulfide linkage similar to that we report here with a binding energy of 98 kJ/mol, while other amino acids had much weaker interactions <4.3 kJ/mol. Our results on CuS are consistent with this, with overall adsorption energies of ∼−1.5 eV or approximately 140 kJ/mol for cysteine on CuS (001). Prior studies of thiols binding to gold surfaces have also reported formation of disulfides in a different motif, in which two adjacent surface-bound alkanethiols join to form a S–S bond;^74^ however, the disulfide formed on gold has both S atoms bound to the surface and is therefore structurally and chemically distinct from that we describe here.

Our studies show that while dissolution of CuS is relatively slow, adsorption of glutathione further reduces this process, even in matrices with relatively high concentrations of small acids that are able to chelate Cu^2+^. These dissolution studies further support the favorable formation of disulfide bond formation in matrices more complex than deionized water. While most environmentally relevant matrices are highly complex, our work suggests that the presence of even low concentrations of glutathione and related thiols may be sufficient to control the formation of molecular coronas in more complex environments, such as in the xylem or phloem of plants or in other environmentally relevant matrices. This work also suggests that CuS has the potential to sequester glutathione, cysteine, and other thiol-containing molecules from complex environments; the associated reduction in concentration of these thiol-containing molecules represents another potential pathway for biological impact of CuS nanoparticles. Glutathione contributes to a number of critical functions in plants, including stress signaling and communication,^22,75^ removal of reactive oxygen species (ROS),^76^ and the chelation of toxic heavy metals.^77^

While this study was motivated by a desire to understand the fundamental surface chemistry associated with Cu-containing nanoparticles underlying their biological impact, the chemically specific formation of molecule–surface bonds has potential impact in other areas. Our studies show that while the disulfide bridges are stable under ordinary conditions even in the presence of small organic acids, the molecule–surface bond can be cleaved through introduction of 2-mercaptoethanol. The ability to form chemically specific molecular layers than can be selectively cleaved under mild conditions through the use of specific chemical reactions (e.g., reduction of the disulfide bridge) suggests many possible applications in more advanced chemical manipulation of CuS surfaces.

## Supporting Information Available

Analysis of XPS data; comparison of X-ray diffraction spectra with reference compounds; Raman spectra of CuS nanoparticles and references compounds; high-resolution XPS spectra; time-resolved FTIR spectra of CuS exposed to glutathione; infrared spectra of molecules in water; details of density functional calculations; calculated surface vibrational modes (PDF)
