# Supplementary material for: Selective Adsorption of Thiol-Containing Molecules on Copper Sulfide Surfaces via Molecule–Surface Disulfide Bridges
Source: J Phys Chem C Nanomater Interfaces. 2025 Jan 17;129(4):1976–87. doi: 10.1021/acs.jpcc.4c06463 (PMC11789770; doi:10.1021/acs.jpcc.4c06463)
Supplement: Supplementary file 1 — jp4c06463_si_001.pdf [file jp4c06463_si_001.pdf]

Supporting Information

Selective Adsorption of Thiol-containing Molecules on Copper Sulfide Surfaces via Molecule-Surface Disulfide Bridges

Connor R. Protter[1], Jennifer L. Bjorklund[2], Sara E. Mason[2], Robert J. Hamers[1*]

[1]Department of Chemistry, University of Wisconsin-Madison, 1101 University Avenue, Madison, WI 53706, USA

[2]Center for Functional Nanomaterials, Brookhaven National Laboratory, Upton, NY 11973, USA

*Corresponding author: rjhamers@wisc.edu

Table of Contents

Characterization of CuO nanoparticles	S2
Figure S1.	S2
Analysis of XPS data	S2
XRD of additional structures	S3
Figure S2.	S3
High-resolution XPS of bare CuS nanoparticles	S4
Table S1.	S4
Figure S3.	S5
Raman spectra of CuS nanoparticles	S5
Figure S4.	S5
High resolution N(1s) XPS spectra of exposed CuS nanoparticles	S6
Figure S5.	S6
Time-resolved FTIR spectra for adsorption/rinse of glutathione on CuS surface	S7
Figure S6.	S7
ATR of biomolecules in water	S8
Figure S7.	S8
Figure S8.	S8
Ab initio Thermodynamics: Surface Stability	S9
Figure S9.	S11
DFT + Thermodynamics: Surface Functional Groups	S12
Adsorption Energy Reaction Schemes	S14
Figure S10.	S14
Figure S11.	S15
Calculated surface vibrational modes	S16
Table S1.	S16

Characterization of CuO nanoparticles

Nanoparticles used in this study had a zeta-potential of -19.7±0.3 mV and a BET surface area of 13.2 m^2/g. Figure S1 shows representative SEM of these nanoparticles.

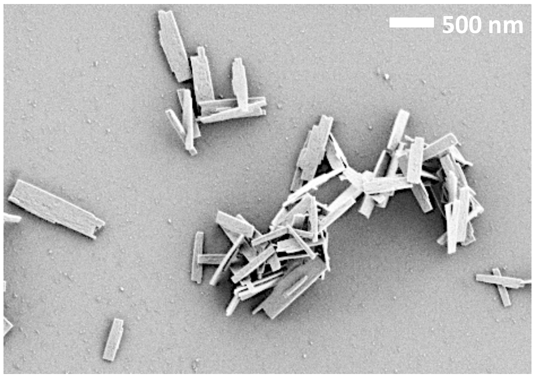

Figure S1. Representative electron micrograph of CuO nanosheets.

Analysis of XPS data

We conducted XPS analysis using Shirley backgrounds and Voigt functions in CasaXPS software.[1] We obtained the composition of the near-surface region using Cu:S= $\frac{\frac{A_{Cu}}{S_{Cu}\lambda_{Cu,CuS}}}{\frac{A_S}{S_S\lambda_{S,CuS}}}$ where A_x is the area of the relevant XPS peak, S_X refers to the atomic sensitivity factor for each element, and λ_x, CuS is the inelastic mean free path of electrons of the respective XPS features. The sensitivity factors used here for surface quantification are $SF_{N(1s)}$=1.68, $SF_{C(1s)}$=1, $SF_{S(2p)}$=1.88, and $SF_{Cu(2p3/2)}$=18.15. These sensitivity factors are specific to the Thermo K-Alpha instrument used for analysis. Inelastic mean free paths used here are for electrons from the element of interest through the CuS substrate, $\lambda_{N(1s)}$=1.968 nm, $\lambda_{S(2p)}$=2.379 nm, and $\lambda_{Cu(2p3/2)}$=1.180 nm. Quantification of nitrogen surface coverage was calculated using the following equation:

$$\text{Coverage}=\frac{A_{N1s}}{A_{Cu2p3/2}}\times\frac{SF_{Cu2p3/2}}{SF_{N1s}}\times\rho_{CuS,\ CuO}\times\lambda_{CuS,CuO}\times\cos\theta$$

Here, A refers to the area of each peak, SF refers to the sensitivity factor of the area being analyzed, and ρ describes the atomic number density of Cu within the bulk material. The inelastic mean free path, λ, of CuS (described above) and CuO (1.997 nm) were determined via the Inelastic-Mean-Free-Path Database using TPP-2M predictive formulae, with stoichiometric ratios for CuS and CuO of 1:1 and Nv=17 for each. The mass densities and bandgaps are 4.68 g/cm^3 and 1.55 eV for CuS, with 6.31 g/cm^3 and 1.2 eV for CuO. Binding energies were calibrated to adventitious carbon in the C1s region at 284.8 eV, which matches the recommended values used in the NIST XPS database.[2]

XRD of additional structures

Figure S2 shows XRD of these CuS nanoparticles compared to multiple copper sulfide structures, including covellite[3], chalcocite[4], and digenite[5] demonstrating that these additional structures are not present in the pattern for these nanoparticles.

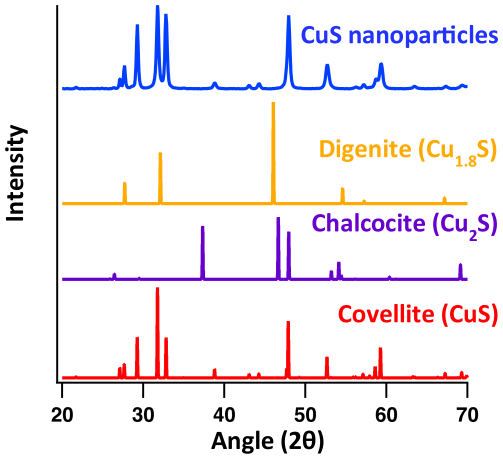

Figure S2. XRD of CuS nanoparticles used here compared to reference copper sulfide diffraction patterns. Reference spectra taken from references 3-5.

High-resolution XPS spectra of bare CuS nanoparticles

Figure S3 shows high resolution Cu(2p) and S(2p) spectra of bare CuS nanoparticles. For the S(2p) region, due to the presence of spin-orbit splitting, each chemically distinct sulfur species gives rise to 2 peaks ($2p_{3/2}$ and $2p_{1/2}$ spin-orbit components), which are in a fixed 2:1 intensity ratio separated by 1.18 eV.

During peak-fitting, the FWHM, energy, and intensity of each of the 3 chemically distinct $2p_{3/2}$ peaks were adjusted to achieve best fit. The fitting was constrained so that each $2p_{1/2}$ component was shifted by 1.18 eV from its corresponding $2p_{3/2}$ component, the FWHM of each $2p_{1/2}$ component was fixed to be the same as its corresponding $2p_{3/2}$ component, and the intensity of each $2p_{1/2}$ component was fixed to be ½ that of its corresponding $2p_{3/2}$ component. The results of the peak-fitting for $2p_{3/2}$ components are summarized below in Table S1.

Table S1. Summary of XPS peak-fitting for $2p_{3/2}$ components.

Binding energy (eV)	FHWM	Area
161.2	0.70	0.097
162.0	0.85	0.34
163.0	2.8	0.56

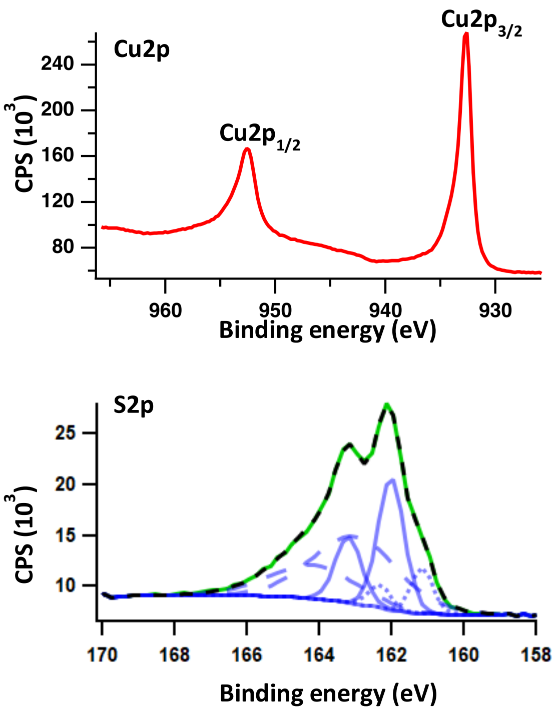

Figure S3. High resolution Cu(2p) and S(2p) spectra of bare CuS nanoparticles. For the S(2p) spectrum, the experimental spectrum is shown in green, with Shirley background and individual fits in blue. The sum of fits is shown as a black dashed line overlaid on the experimental spectrum.

Raman spectra of CuS nanoparticles.

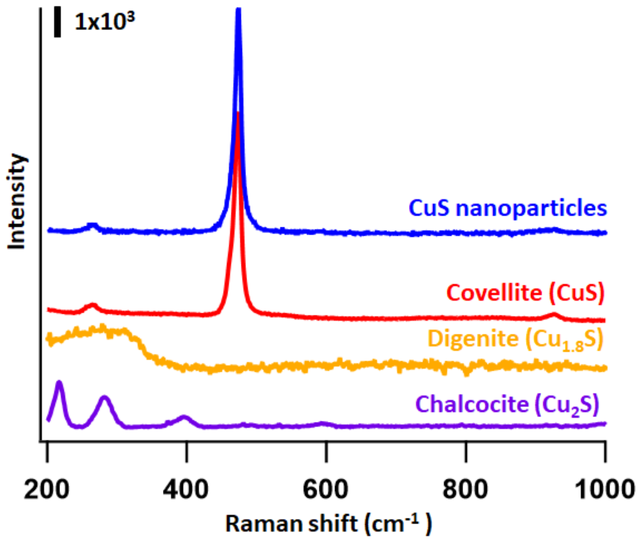

Figure S4. Raman of CuS nanoparticles compared to other stoichiometric and non-stoichiometric copper sulfides. Reference spectra were taken from reference 6

Figure S4 shows Raman of CuS nanoparticles compared to spectra for other stoichiometric and non-stoichiometric copper sulfides.[6] Peaks associated with covellite are present in the spectrum for CuS nanoparticles, while chalcocite and digenite are absent.

High resolution N(1s) XPS spectra of exposed CuS nanoparticles

Figure S5 shows compared high resolution N(1s) spectra for CuS nanoparticles exposed to GSH, CYS, OTA, HAL, GSSG, and GSH followed by a disulfide reducing agent. These data have been divided by the relative area of the Cu($2p_{3/2}$) peak used for determining their surface coverage, in order to account for fluctuations in instrument data collection, spots on each sample, and the emission intensity of the substrate.

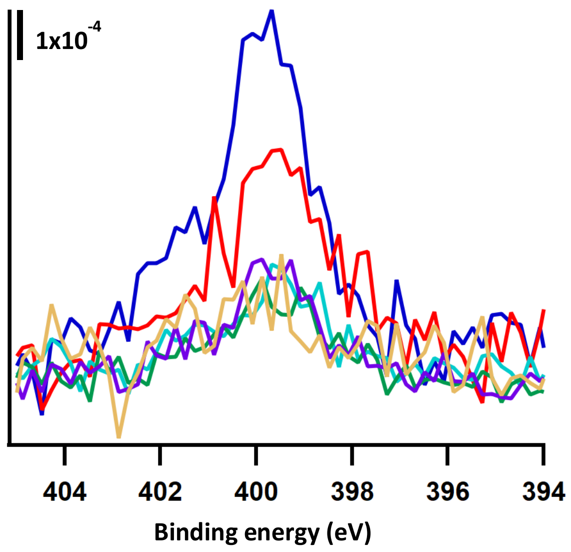

Figure S5. Comparison of high resolution N1s spectra for biomolecule exposures, normalized to Cu2p regions used in quantification

Time-resolved FTIR spectra for adsorption/rinse of glutathione on CuS surface

Figure S6 shows a typical set of time-resolved spectra for the adsorption and rinse of glutathione on the surface of CuS over the course of these experiments. As described earlier, these data are background-corrected relative to the end of the pure water equilibration step. Each spectrum takes approximately 4 minutes to collect. The spectra with solid lines are during the adsorption flow step, with peaks growing in that are representative of glutathione in solution. After rinsing, shown with dashed lines, the peaks and their relative position and intensity have not significantly changed.

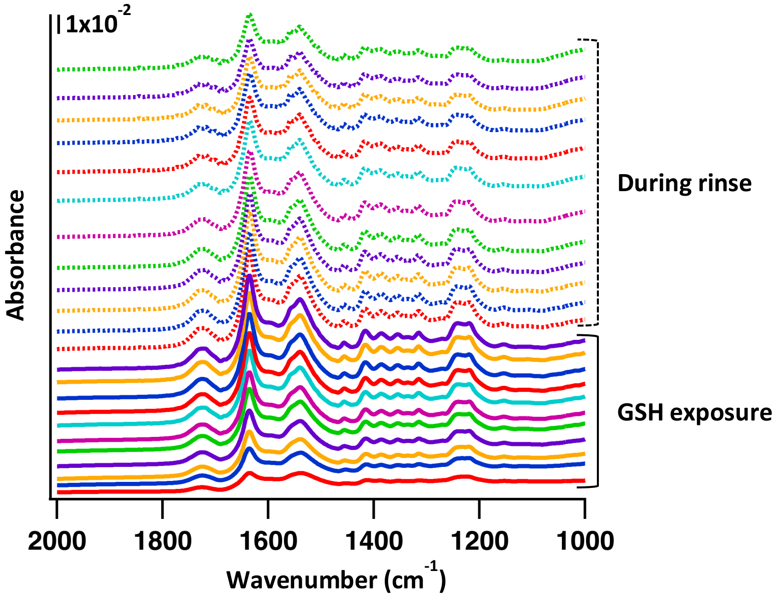

Figure S6. Representative time-resolved spectra for glutathione on CuS.

ATR of biomolecules in water

Figure S7 shows ATR-FTIR of these biomolecules in water at a concentration of 100 mM. These spectra show that all biomolecules used here absorb similar amounts of infrared light to each other and provide structures for comparison to other ATR data analyzed here.

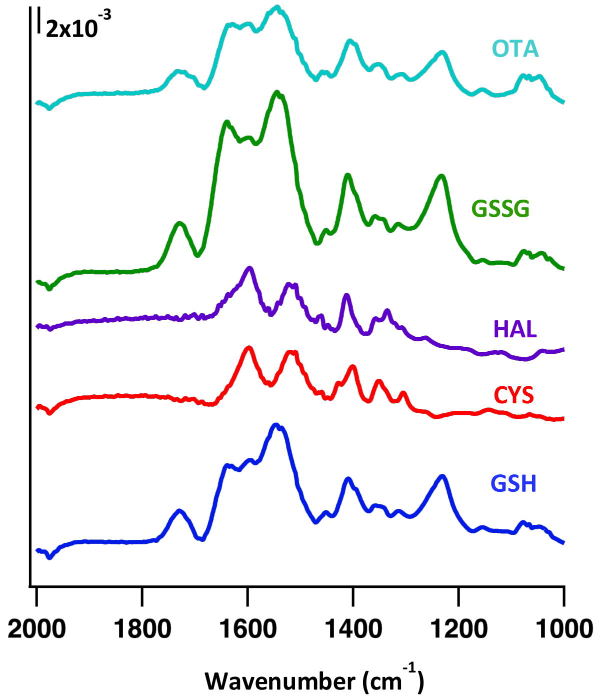

Figure S7. ATR-FTIR of dissolved biomolecules used in this study at 100 mM

Figure S8 shows infrared spectra of each biomolecule after being flowed over a germanium ATR element for 90 minutes, followed by 90 minutes of rinsing.

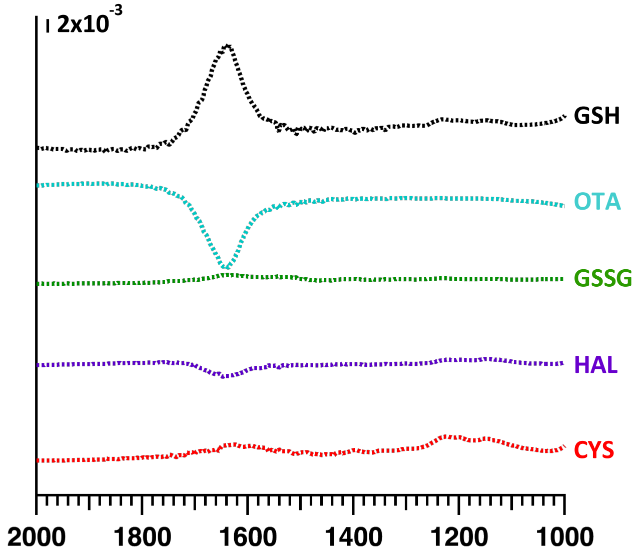

Figure S8. Infrared spectra of each biomolecule after 90 minutes of flow and 90 minutes of rinse over bare Ge element at 65 μM.

Ab initio Thermodynamics: Surface Stability

Ab initio atomistic thermodynamics[7-10] was used to predict surface free energies. Surface free energies in vacuum were calculated as a function of the chemical potential:

$$\gamma(T,p) = \frac{1}{2A}\left[G - \sum_i N_i \mu_i(T,p)\right] \quad \text{(S1)}$$

where G refers to the Gibbs free energy of the relaxed slab model, $2A$ refers to the surface area of the two equivalent slabs, μ_i(T,p) is the chemical potential of species i, and N_i is the number of atoms of species i. Values of Gibbs free energy were obtained by incorporating the vibrational zero point energy in the DFT total energy; entropic contributions to the total energy are negligible under the presumed conditions. The surface free energy approach of Reuter and Scheffler is well-documented in other work and is summarized for the CuS system of interest here. The expression for γ(T,p) can be rewritten as:

$$\gamma(T,p) = \frac{1}{2A}[G_{slab}^{CuS} - N_{Cu}\mu_{Cu}(T,p) - N_S\mu_S(T,p)] \quad \text{(S2)}$$

where G_{slab}^{CuS} is the Gibbs free energy of the CuS surface slab with two equivalent surfaces. The free energy of the bulk CuS (G_{bulk}^{CuS}) can be used to rewrite Equation S2 so that it is a function of μ_S:

$$(T,p) = \frac{1}{2A}[G_{slab}^{CuS} - N_{Cu}G_{bulk}^{CuS} + (N_{Cu} - N_S)\mu_S(T,p)] \quad \text{(S3)}$$

It is important to consider the thermodynamically accessible range for the sulfur chemical potential. The S-poor limit can be defined as the chemical potential from bulk CuS, while the S-rich limit is defined as the chemical potential from bulk α-S, as done in previous work.[11]

We assessed the surface free energy at 0 K to determine the surface stability under ultrahigh vacuum without temperature effects. The five bare surfaces exhibit different numbers (*N*) of Cu and S atoms, which impacts the slopes of the lines shown in Figure S9. The two surfaces that do not have constant surface free energies are the Cu T and SS surfaces, where Cu T has *N*(Cu) > *N*(S) and SS has *N*(S) > *N*(Cu). As expected, these two surfaces have equal and opposite slopes as a function of μ_S.

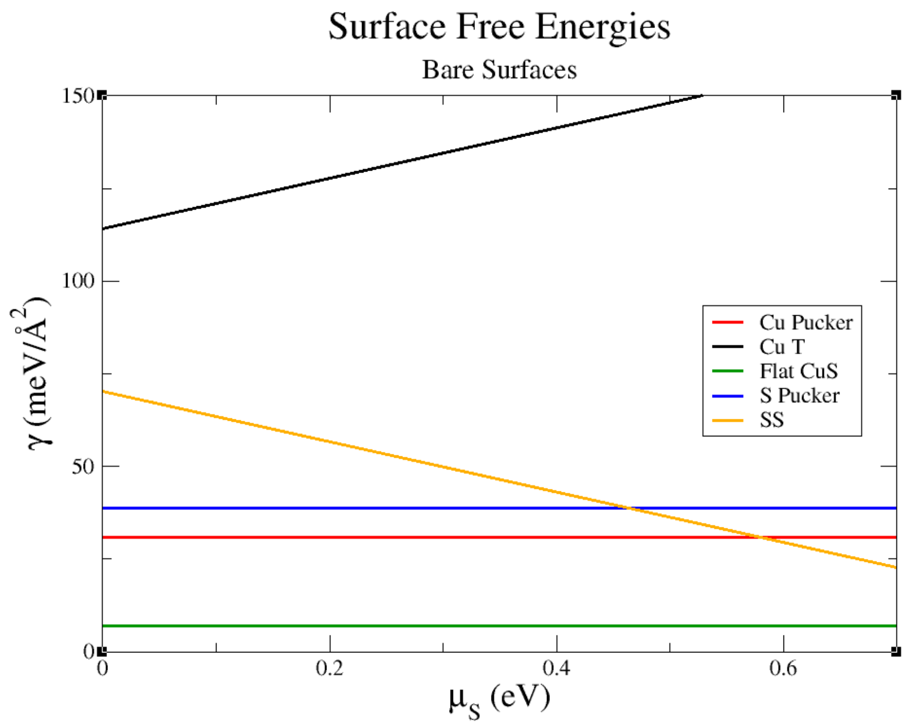

Figure S9. Plot of γ, the surface free energy of the surface terminations shown in Figure 7, with respect to μ_s, the chemical potential of S. The lefthand side of the plot corresponds to the S-poor environment and the right corresponds to the S-rich environment.

Under S-rich conditions, the trend in increasing surface stability is: Cu T < S Pucker < Cu Pucker < SS < Flat CuS. As the conditions change to the S-poor regime, the SS surface becomes increasingly less stable, while the Cu T surface becomes slightly more stable. The trends in surface free energy agree well with previous findings on the bare CuS surface terminations, with the Flat CuS being the lowest energy structure and the Cu T being the highest.[11, 12]

DFT + Thermodynamics: Surface Functional Groups

Under aqueous conditions undercoordinated S and Cu atoms will react with nearby species such as water and water products to regain coordinative saturation. Calculations assumed

that bulk-terminated surfaces exposing under-coordinated Cu atoms were hydroxylated to form CuOH surface functional groups and surfaces exposing under-coordinated S atoms were protonated to form SH functional groups.

The thermodynamics of surface functional group formation were assessed using the DFT solvent-ion approach, which approximates the free energy change associated with the dissolution or deposition of surface atoms and the adsorption or desorption of H_aO_b species (H, OH, O, and H_2O). The method requires a reference surface structure and the partitioning of the overall process into elementary steps. The contribution of each step to the overall change in Gibbs free energy is determined either using DFT total energies or tabulated experimental data regarding the aqueous electrochemical properties and with analytical corrections to account for changes from standard conditions.[13] This approach has been applied to a variety of systems and applications- from predicting the metal release or dissolution from battery cathode materials[14-17] to understanding the sorption preferences of contaminants at the mineral-water interface.[18, 19] This method has also been applied toward understanding how nanomaterials can transform under aqueous conditions.[20, 21]

The utility of this model allows the overall modeled reaction to be partitioned into purely-DFT components (ΔG_1) and components determined using experimental information (ΔG_2). For example, consider the formation of SH functional groups on the S-terminated surface, where the overall reaction is shown in Equation (S4):

$$\text{CuS-Cu} + H_2O_{(l)} \rightarrow \text{CuS-CuOH} + H^+_{(aq)} \quad \text{(S4)}$$

This reaction is broken down into elementary steps that can be descried either using DFT or tabulated experimental information. ΔG_1 is calculated using DFT total energies and Hess's Law according to Equation (S5), where the reactants are referenced to their standard states ($H_{2\,(g)}$ and

$O_{2\,(g)}$). DFT total energies are converted to values of *G* through the inclusion of zero-point energy and TΔS corrections.

$$\text{CuS-Cu} + \tfrac{1}{2}\,H_{2\,(g)} + \tfrac{1}{2}\,O_{2\,(g)} \rightarrow \text{CuS-CuOH} \quad \text{(S5)}$$

The aqueous chemistry is described in ΔG_2, which is calculated using a variation of the Nernst equation shown in Equation (S6) that allows for the incorporation of key experimental parameters such as concentration, temperature, and solution pH.

$$\Delta G_2 = \Delta G^{\circ}_{\mathrm{SHE}} - n_e(eU_{\mathrm{SHE}}) - 2.303 n_{H^+} k_{\mathrm{B}} T \mathrm{pH} + k_{\mathrm{B}} T ln\ a_{H_xO_y} \quad \text{(S6)}$$

In Equation (S6), $\Delta G^{\circ}_{\mathrm{SHE}}$ refers to the Gibbs free energy of hydrated ion formation with respect to their ground state referenced to the standard hydrogen electrode (SHE), U_{SHE} is the applied external potential (assumed to be 0 V in this work), $a_{H_xO_y}$ is the activity of the constituent ions (taken to be 1×10^{-6} M). The n_{H^+} and n_e terms refer to the numbers of protons and electrons, respectively, in the elementary steps to describe the formation of surface functional groups. ΔG_2 values are computed referencing thermodynamic information according to the each of the electrochemical reactions in Equations (S7) and (8S).

$$H^+_{(aq)} + 1\,e^- \rightarrow \tfrac{1}{2}\,H_{2\,(g)} \quad \text{(S7)}$$

$$H_2O_{(l)} \rightarrow 2\,H^+_{(aq)} + \tfrac{1}{2}\,O_{2\,(g)} \quad \text{(S8)}$$

The ΔG_1 and ΔG_2 terms are summed up to calculate ΔG_{total} for the formation of each surface functional group. In the case of forming SH functional groups on S-terminated surfaces, the ΔG_2 term only relies on Equation (S7) to account for aqueous protons. These reactions can be easily modified to describe the formation of different surface functional groups on other materials.[11, 12]

Adsorption Energy Reaction Schemes

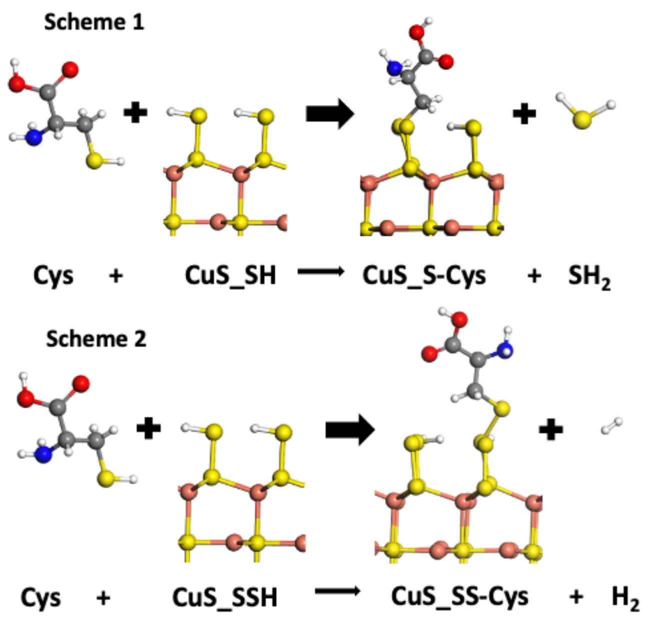

Figure S10. Ball-and-stick representations of the two possible reaction schemes used to compute E_{ads}, where Scheme 1 results in the formation of S-S bonds and an SH_2 molecule and Scheme 2 forms S-S-S bonds and an H_2 molecule.

There are two possible reaction mechanisms that were considered, shown in Figure S10. The first reaction (Scheme 1) is applied to both the S Pucker and SS structures and involves a ligand exchange reaction with a surface SH group and the SH group of cysteine, producing a SH_2 molecule. The second reaction (Scheme 2) applies only to the SS structure and assumes that the product is a H_2 molecule and that S-S-S bonds bind the cysteine to the CuS surface. Adsorption energies (E_{ads}) are calculated using Hess's Law for each of the schemes in Figure S10, where the products are the adsorption complex and a SH_2/H_2 gas molecule and the reactants are the hydrated surface and the cysteine molecule.

The surface free energies of the S-terminated structures that form SH functional groups were also calculated are compared in Figure S11. The chemical potential of H is referenced to $H_{2\,(g)}$. This approach compares well to the DFT + Thermodynamics approach that references the

source of protons to be from the splitting of $H_{2\,(g)}$. We note that the value of γ for the bare Flat CuS surface was approximately 7 meV/Å^2; upon protonation, is destabilized by 30 meV/Å^2. For the protonated SS and S Pucker surfaces, both become more stable than their bare counterparts. Under S-rich conditions, the SS surface becomes 36 meV/Å^2 more stable, while protonation of the S Pucker surface becomes 51 meV/Å^2 lower in energy.

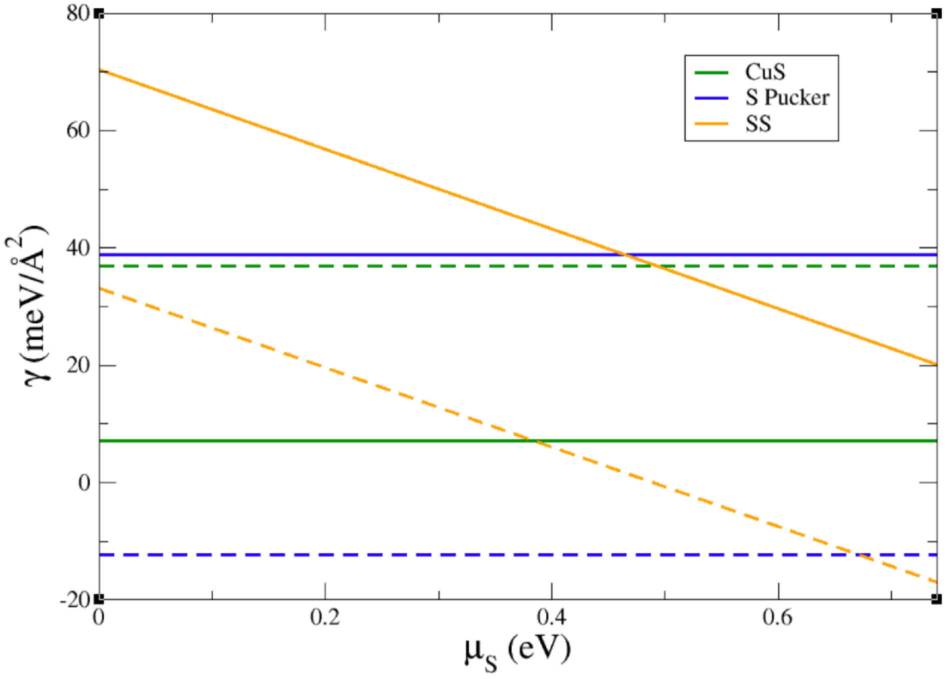

Figure S11. Surface free energies of the three bare (solid lines) and SH terminated (dashed lines) surfaces as a function of μ_S.

Calculated surface vibrational modes

Table S2.

	Without Cysteine		With Cysteine	
Surface	Bulklike S-S Stretch	Surface S-S Stretch	Bulklike S-S Stretch	Surface S-S Stretch
SSH	189, 426	443, 452	189, 414, 415, 416, 426	403, 436, 437, 439, 448, 469
SH	190, 422		189, 408, 410, 412, 422	212, 439

DFT calculated vibrational modes for the SSH and SH terminated surfaces both show bulklike interior S-S stretching modes at approximately 189 and 422-426 cm^{-1}, with the SSH terminated surface also having S-S vibrations for the exposed surface atoms at 443 and 452 cm^{-1}. When the cysteine adsorbate is present in the 2x2 supercell, these modes spread out into multiple degenerate modes. For example, the SH terminated pristine surface has a bulklike S-S stretch at 422 cm^{-1} that separates into modes ranging from 408 – 422 cm^{-1}. Similar observations are drawn regarding the bulklike S-S stretches for the SSH terminated surface. However, the two surface S-S modes for the SSH terminated surface spread out more significantly (403-469 cm^{-1}), where the mode at 403 cm^{-1} corresponds only to the S-S bond that connects the cysteine adsorbate to the surface and the rest are assigned to the S-SH functional groups. Considering collectively the pristine and cysteine-complexed surfaces, the bulklike S-S stretches tend to occur at slightly lower frequencies (408-426 cm^{-1}) compared to surface S-S stretches (436-469 cm^{-1}). Signature S-S vibrations between the surface and the cysteine adsorbate are computed to be 439 cm^{-1} (SH terminated surface) and 403 cm^{-1} (S-SH terminated surface).

Studies have shown that DFT predictions of the exact location of certain vibrational features are sensitive to calculation parameters such as functional and [22] as well as the employment of different solvation models.[23] The predicted S-S vibration here are lower than expected the expected range of 500-600 cm^{-1}, which have been reported for organic compounds, peptides, and proteins.[22, 24-27] It is possible that S-S bond formation at the nanoparticle surface may have shifted vibrational modes compared to those reported for molecular and biomolecular structures. One possible remedy to the low frequencies is to apply a scaling factor to systematically increase the frequencies. However, selecting the appropriate scaling factor is a challenge without

extensive benchmarking due to its dependence on the calculation parameters and system specifics. [28-30].

(1) Fairley, N.; Fernandez, V.; Richard-Plouet, M.; Guillot-Deudon, C.; Walton, J.; Smith, E.; Flahaut, D.; Greiner, M.; Biesinger, M.; Tougaard, S.; et al. Systematic and collaborative approach to problem solving using X-ray photoelectron spectroscopy. *Applied Surface Science Advances* **2021**, *5*, 100112. DOI: https://doi.org/10.1016/j.apsadv.2021.100112.

(2) NIST X-ray Photoelectron Spectroscopy Database, NIST Standard Reference Database Number 20, , Gaithersburg MD, 20899 (2000), National Institute of Standards and Technology.

(3) Berry, L. G. The crystal structure of covellite, CuSe and klockmannite, CuSe. *American Mineralogist* **1954**, *39* (5-6), 504-509. (acccessed 4/19/2024).

(4) Buerger, M. J.; Wuensch, B. J. Distribution of Atoms in High Chalcocite, Cu_2S. *Science* **1963**, *141* (3577), 276-277. DOI: 10.1126/science.141.3577.276 (acccessed 2024/04/25).

(5) Morimoto, N.; Kullerud, G. Polymorphism in digenite. *American Mineralogist* **1963**, *48* (1-2), 110-123.

(6) Lafuente, B.; Downs, R. T.; Yang, H.; Stone, N. 1. The power of databases: The RRUFF project. In *Highlights in Mineralogical Crystallography*, Thomas, A., Rosa Micaela, D. Eds.; De Gruyter (O), 2016; pp 1-30.

(7) Reuter, K.; Scheffler, M. First-Principles Atomistic Thermodynamics for Oxidation Catalysis: Surface Phase Diagrams and Catalytically Interesting Regions. *Physical Review Letters* **2003**, *90* (4), 046103. DOI: 10.1103/PhysRevLett.90.046103.

(8) Reuter, K.; Stampf, C.; Scheffler, M. AB Initio Atomistic Thermodynamics and Statistical Mechanics of Surface Properties and Functions. In *Handbook of Materials Modeling: Methods*, Yip, S. Ed.; Springer Netherlands, 2005; pp 149-194.

(9) Rogal, J.; Reuter, K. Ab initio atomistic thermodynamics for surfaces: A primer. In *experiment, modeling and simulation of gas-surface interactions for reactive flows in hypersonic flights*, Vol. 14; NATO 2007; pp 2-1.

(10) McHale, J. M.; Auroux, A.; Perrotta, A. J.; Navrotsky, A. Surface Energies and Thermodynamic Phase Stability in Nanocrystalline Aluminas. *Science* **1997**, *277* (5327), 788-791. DOI: doi:10.1126/science.277.5327.788.

(11) Morales-García, Á.; He, J.; Soares, A. L.; Duarte, H. A. Surfaces and morphologies of covellite (CuS) nanoparticles by means of *ab initio* atomistic thermodynamics. *CrystEngComm* **2017**, *19* (22), 3078-3084, DOI: 10.1039/C7CE00203C.

(12) Soares Jr, A. L.; Dos Santos, E. C.; Morales-García, Á.; Duarte, H. A.; De Abreu, H. A. The Stability and Structural, Electronic and Topological Properties of Covellite (001) Surfaces. *ChemistrySelect* **2016**, *1* (11), 2730-2741. DOI: https://doi.org/10.1002/slct.201600422 .

(13) Rong, X.; Kolpak, A. M. Ab Initio Approach for Prediction of Oxide Surface Structure, Stoichiometry, and Electrocatalytic Activity in Aqueous Solution. *The Journal of Physical Chemistry Letters* **2015**, *6* (9), 1785-1789. DOI: 10.1021/acs.jpclett.5b00509.

(14) Bennett, J. W.; Jones, D.; Huang, X.; Hamers, R. J.; Mason, S. E. Dissolution of Complex Metal Oxides from First-Principles and Thermodynamics: Cation Removal from the (001) Surface of $Li(Ni_{1/3}Mn_{1/3}Co_{1/3})O_2$. *Environmental Science & Technology* **2018**, *52* (10), 5792-5802. DOI: 10.1021/acs.est.8b00054.

(15) Bennett, J. W.; Jones, D. T.; Hamers, R. J.; Mason, S. E. First-Principles and Thermodynamics Study of Compositionally Tuned Complex Metal Oxides: Cation Release from the (001) Surface of Mn-Rich Lithium Nickel Manganese Cobalt Oxide. *Inorganic Chemistry* **2018**, *57* (21), 13300-13311, Article. DOI: 10.1021/acs.inorgchem.8b01855.
(16) Buchman, J. T. T.; Bennett, E. A. A.; Wang, C. Y.; Tamijani, A. A.; Bennett, J. W. W.; Hudson, B. G. G.; Green, C. M. M.; Clement, P. L. L.; Zhi, B.; Henke, A. H. H.; et al. Nickel enrichment of next-generation NMC nanomaterials alters material stability, causing unexpected dissolution behavior and observed toxicity to *S. oneidensis* MR-1 and D. magna. *Environ.-Sci. Nano* **2020**, *7* (2), 571-587, DOI: 10.1039/c9en01074b.
(17) Hudson, B. G.; Jones, D. T.; Rivera Bustillo, V. M.; Bennett, J. W.; Mason, S. E. Understanding the Mechanism of Secondary Cation Release from the (001) Surface of $Li(Ni_{1/3}Mn_{1/3}Co_{1/3})O_2$: Insights from First-Principles. *The Journal of Physical Chemistry C* **2023**, *127* (43), 21022-21032. DOI: 10.1021/acs.jpcc.3c02764.
(18) Tamijani, A. A.; Bjorklund, J. L.; Augustine, L. J.; Catalano, J. G.; Mason, S. E. Density Functional Theory and Thermodynamics Modeling of Inner-Sphere Oxyanion Adsorption on the Hydroxylated α-Al_2O_3(001) Surface. *Langmuir* **2020**, *36* (44), 13166-13180. DOI: 10.1021/acs.langmuir.0c01203.
(19) Augustine, L. J.; Abbaspour Tamijani, A.; Bjorklund, J. L.; Al-Abadleh, H. A.; Mason, S. E. Adsorption of small organic acids and polyphenols on hematite surfaces: Density Functional Theory + thermodynamics analysis. *Journal of Colloid and Interface Science* **2022**, *609*, 469-481. DOI: https://doi.org/10.1016/j.jcis.2021.11.043.
(20) Huang, X.; Bennett, J. W.; Hang, M. N.; Laudadio, E. D.; Hamers, R. J.; Mason, S. E. Ab Initio Atomistic Thermodynamics Study of the (001) Surface of LiCoO2 in a Water Environment

and Implications for Reactivity under Ambient Conditions. *The Journal of Physical Chemistry C* **2017**, *121* (9), 5069-5080. DOI: 10.1021/acs.jpcc.6b12163.

(21) Grimes, R. T.; Leginze, J. A.; Zochowski, R.; Bennett, J. W. Surface Transformations of Lead Oxides and Carbonates Using First-Principles and Thermodynamics Calculations. *Inorganic Chemistry* **2021**, *60* (2), 1228-1240. DOI: 10.1021/acs.inorgchem.0c03398.

(22) Wong, M. W. Vibrational frequency prediction using density functional theory. *Chemical Physics Letters* **1996**, *256* (4), 391-399. DOI: https://doi.org/10.1016/0009-2614(96)00483-6.

(23) Gwee, E. S. H.; Seeger, Z. L.; Appadoo, D. R. T.; Wood, B. R.; Izgorodina, E. I. Influence of DFT Functionals and Solvation Models on the Prediction of Far-Infrared Spectra of Pt-Based Anticancer Drugs: Why Do Different Complexes Require Different Levels of Theory? *ACS Omega* **2019**, *4* (3), 5254-5269. DOI: 10.1021/acsomega.8b03455.

(24) Bastian, E. J., Jr.; Martin, R. B. Disulfide vibrational spectra in the sulfur-sulfur and carbon-sulfur stretching region. *The Journal of Physical Chemistry* **1973**, *77* (9), 1129-1133. DOI: 10.1021/j100628a010.

(25) Han, S.-L.; Rivier, J. E.; Scheraga, H. A. Conformational studies of somatostatin and selected analogues by Raman spectroscopy. *International Journal of Peptide and Protein Research* **1980**, *15* (4), 355-364. DOI: https://doi.org/10.1111/j.1399-3011.1980.tb02912.x.

(26) Sugeta, H.; Go, A.; Miyazawa, T. S–S and C–S stretching vibrations and molecular conformations of dialkyl disulfides and cystine. *Chemistry Letters* **2006**, *1* (1), 83-86. DOI: 10.1246/cl.1972.83.

(27) Hernández, B.; Pflüger, F.; López-Tobar, E.; Kruglik, S. G.; Garcia-Ramos, J. V.; Sanchez-Cortes, S.; Ghomi, M. Disulfide linkage Raman markers: a reconsideration attempt. *Journal of Raman Spectroscopy* **2014**, *45* (8), 657-664. DOI: https://doi.org/10.1002/jrs.4521.

(28) Scott, A. P.; Radom, L. Harmonic Vibrational Frequencies: An Evaluation of Hartree−Fock, Møller−Plesset, Quadratic Configuration Interaction, Density Functional Theory, and Semiempirical Scale Factors. *The Journal of Physical Chemistry* **1996**, *100* (41), 16502-16513. DOI: 10.1021/jp960976r.

(29) Merrick, J. P.; Moran, D.; Radom, L. An Evaluation of Harmonic Vibrational Frequency Scale Factors. *The Journal of Physical Chemistry A* **2007**, *111* (45), 11683-11700. DOI: 10.1021/jp073974n.

(30) Alecu, I. M.; Zheng, J.; Zhao, Y.; Truhlar, D. G. Computational Thermochemistry: Scale Factor Databases and Scale Factors for Vibrational Frequencies Obtained from Electronic Model Chemistries. *Journal of Chemical Theory and Computation* **2010**, *6* (9), 2872-2887. DOI: 10.1021/ct100326h.
